# Chemical Upcycling of Nitrile Butadiene Rubbers to Polyamines and Polyols by Chemoselective Catalytic Hydrogenation

**DOI:** 10.1002/anie.202525705

**Published:** 2026-03-18

**Authors:** Alejandra Sophia Lozano Perez, Raymundo Marcial‐Hernandez, Harini Sampathkumar, Oluchi Emenike, Ketan Pancholi, Claire N. Brodie, Daniel M. Dawson, Amit Kumar

**Affiliations:** ^1^ EaStCHEM, School of Chemistry University of St Andrews St Andrews UK; ^2^ School of Computing, Engineering and Technology, The Sir Ian Wood Building Robert Gordon University Aberdeen UK

**Keywords:** nitrile butadiene rubber, hydrogenation, polyamine, polyol, ruthenium

## Abstract

We report here two new approaches for the chemical recycling/upcycling of nitrile butadiene rubber (NBR) to make either polyamines or polyols. Both processes are achieved through ruthenium‐catalyzed hydrogenation reactions, where the chemoselective reduction of nitriles leads to the formation of either amines or alcohols. The hydrogenation of NBR to polyamines could be achieved at temperatures as low as 35°C, whereas a higher temperature (150°C) was required for the formation of polyols with catalytic turnover numbers reaching up to 2000. Additionally, polyamines were demonstrated for their potential application in CO_2_ capture, absorbing 1.34 mmol of CO_2_/g of absorbents. This was significantly higher in comparison to that obtained in the case of NBR, which absorbed only 0.015 mmol of CO_2_/g of absorbent. The synthesized polyol exhibited a markedly greater ductility than the commercial NBR, reaching an elongation at break of ≈ 550% versus ≈ 420% for NBR, suggestive of potential use in stretch‐demanding applications.

## Graphical abstract

We demonstrate here the transformation of NBR (Nitrile butadiene rubber) including post‐consumer waste sourced from nitrile gloves and o‐rings into polyamines and polyols via ruthenium catalyzed hydrogenation.

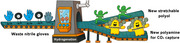

## 1 Introduction

The development of sustainable methods for the upcycling of plastic waste is one of the most important challenges in achieving a circular economy. Among various plastics that need to be recycled and are subject to continuous study, nitrile butadiene rubber (NBR) has received comparatively little attention despite a large market (36 million tons or USD 2.5 billion globally per year) [1] and wide applications ranging from disposable gloves to hoses, seals, and O‐rings. It is noteworthy that the recycling of NBR is challenging due to its thermoset nature, with less than 2% currently recycled [2], often through low‐value downcycling. Sustainable chemical recycling or upcycling routes to convert NBR into valuable chemicals or materials would be a huge leap towards a circular economy.

**FIGURE 1:**
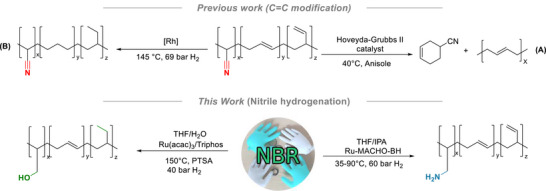
NBR modifications based on the transformation of C═C bonds (previously reported NBR metathesis (A) and double bond hydrogenation (B)) and present work on nitrile hydrogenation.

Most of the previously reported strategies for the chemical recycling of NBR are focused on modifying the C═C double bonds via reactions such as olefin metathesis (Figure 1A) [3], hydrogenation (Figure 1B) [4], and epoxidation. For instance, Thomas, Gauvin, and coworkers recently reported cross‐metathesis of NBR to synthesize unsaturated polyesters (Figure 1A) [3]. In contrast, chemical modification of the nitrile groups in NBR has received much less attention, and explored reactions include nucleophilic addition, cycloaddition, reduction, and hydrolysis [5]. Notably, the only method reported in peer‐reviewed literature for reducing nitrile groups in NBR to amines involves lithium aluminium hydride (LiAlH_4_), a reagent that generates significant waste and is not sustainable for large‐scale plastic recycling [6, 7].

Catalytic hydrogenation, in comparison, is a greener and more atom‐economical method for organic transformations [8]. This approach has recently been applied to the chemical recycling of polymers such as polyesters [8, 9, 10], nylons [11], polycarbonates [12, 13, 14], polyurethanes [15], and polyureas [16]. However, to our knowledge, no report exists on the catalytic hydrogenation of NBR for upcycling purposes in peer‐reviewed literature. We envisioned that the tool of catalytic hydrogenation could be employed to convert NBR into valuable polyamines and polyols. During the preparation of this manuscript, Skrydstrup and co‐workers reported a preprint on the hydrogenation of NBR and styrene–butadiene rubber for the preparation of materials designed for CO_2_ capture [17].

It is noteworthy that there is a growing need for sustainable synthetic routes to polyamines and polyols. Polyamines (global market size: > USD 800 million) [18], for example, are of increasing interest for applications such as CO_2_ capture [19] and gene delivery [20]. Yet the most widely studied polyamines, branched polyethyleneimines, are industrially synthesized from aziridine, a highly toxic and hazardous monomer [21]. Linear polyethyleneimines require a two‐step synthesis involving the ring‐opening polymerization of 2‐ethyl‐2‐oxazoline followed by acidic hydrolysis [22]. The development of new and sustainable methods to make polyamine derivatives is therefore of high importance and will benefit several disciplines. Similarly, polyols, including polyvinyl alcohols and polyethylene glycols, are used in adhesives, dispersants, emulsifiers, and lubricants [23]. These polymers are typically not recycled due to collection challenges, making polyols derived from waste particularly attractive for promoting circularity. In this report, we present the first example of NBR hydrogenation to produce polyamines and polyols—offering promising new routes for the chemical upcycling of nitrile rubber waste into high‐value materials.

## 2 Results and Discussion

**FIGURE 2:**
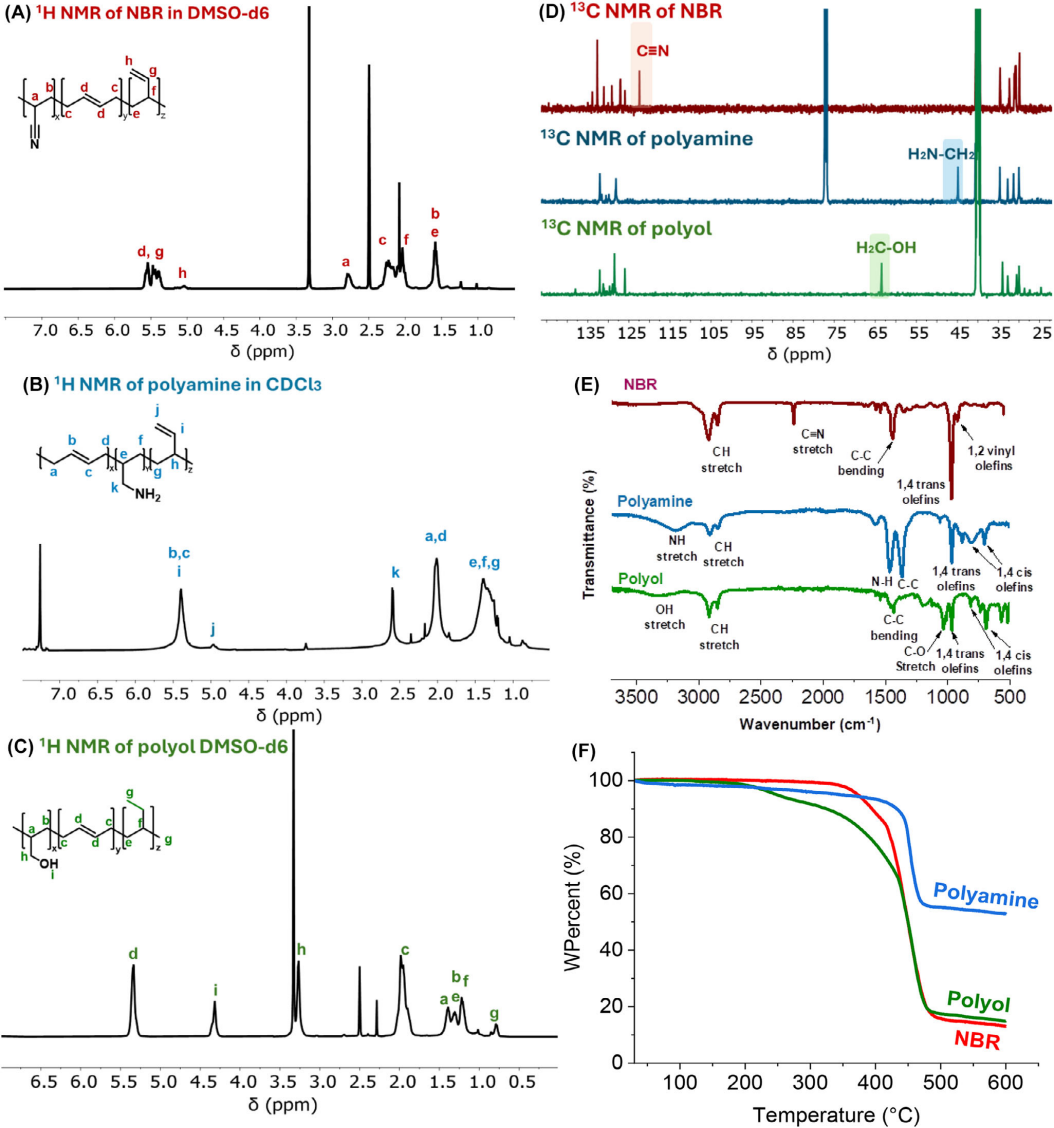
(A) ^1^H NMR spectrum of NBR, (B) polyamine, and (C) polyol. (D) ^13^C{^1^H} NMR spectra of NBR, polyamine and polyol. (E) FT‐IR spectra of NBR, polyamine and polyol. (F) TGA traces of NBR (onset temperature (O.T.) 404°C), polyamine (O.T. 439°C) and polyol (O.T. 409°C). The spectra of NBR correspond to commercially available technical grade materials, while the spectra of the polyamines and polyols correspond to the materials reported in this work (Samples of Table 1, entry 2 and Table 2, entry 3). DSC of NBR (Figure S7), polyamine (Figure S21), and polyol (Figure S69) can be found in the Supporting Information.

We started our investigation by studying the catalytic hydrogenation of NBR using transition‐metal pincer catalysts known for the hydrogenation of polar bonds, in particular nitriles [24, 25, 26, 27, 28, 29, 30, 31, 32, 33, 34, 35]. For example, Beller has reported the use of Ru‐MACHO‐BH (**Ru‐1**), and Ru‐MACHO (**Ru‐2**) complexes for the selective hydrogenation of aliphatic nitriles to amines [36]. Inspired by this report, we tested these complexes for the hydrogenation of NBR. A solvent mixture of THF and *i*PrOH was used based on Beller's report on the hydrogenation of nitriles to amines and for the solubility of NBR [35]. Performing the hydrogenation of NBR using Ru‐MACHO‐BH (1 mol%) at 55°C, and 60 bar H_2_ pressure for 16 h (Table, entry 1) produced a material that was soluble in CHCl_3_ after prolonged stirring for 30 h at room temperature. Analysis of the material by IR and NMR spectroscopy confirmed the formation of a polyamine in 71% yield (Table 1, entry 1). Additionally, the IR analysis of the material showed the appearance of a new signal at ∼1900 cm^−1^, which we attribute to the formation of a C═C═N nitrile‐imine type group [37] leading to the cross‐linking of the polymer chain that could be responsible for the poor solubility of the polymer (Figure S11). Another possibility of cross‐linking could arise from the isomerization of the C═C bond, followed by the C─C bond forming Michael addition reactions as reported by de Vries and Otten for 3‐pentenenitrile [38]. Performing the hydrogenation of polyacrylonitrile under similar conditions also produced a material with a new signal in the IR spectrum at ∼1900 cm^−1^, confirming that the speculated cross‐linking originates from polyacrylonitrile rather than polybutadiene (see ESI, Figure S56). Interestingly, when Ru‐MACHO‐BH (**Ru‐1**, 1 mol%) was used in combination with KO*t*Bu (5 mol%), the hydrogenation reaction under the same conditions produced polyamine in >99% yield, and negligible cross‐linking was observed in this case, possibly due to a higher rate of hydrogenation in the presence of KO*t*Bu (Table 1, entry 2) [39, 40]. NMR and IR spectroscopy confirmed the complete conversion of nitriles to primary amines, without hydrogenating either terminal or internal C═C bonds (Figure 2).

**TABLE 1:** Hydrogenation of NBR into polyamines.^a^

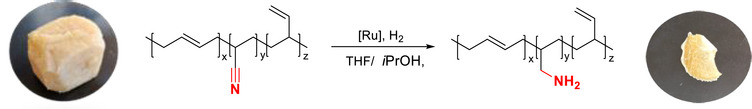								
Entry	Pre‐catalyst	Additive (mol %)	Temp (°C)	Pressure (bar)	Time (h)	Solubility	Conversion (%)^b^	Polyamine Yield (%)^c^
1.	**Ru‐1** (1 mol%)	—	55	60	16	CHCl_3_	75	71^d^
2.	**Ru‐1** (1 mol%)	KO*t*Bu (5%)	55	60	16	CHCl_3_	100	>99
3.	—	KO*t*Bu (5%)	55	60	16	CHCl_3_, THF, DMF	0	0
4.	**Ru‐2** (1 mol%)	KO*t*Bu (5%)	55	60	16	CHCl_3_	100	>99
5.	**Ir‐1** (1 mol%)	KO*t*Bu (5%)	55	60	22	—	—	—
6.	**Ru‐1** (1 mol%)	KO*t*Bu (5%)	90	60	3	CHCl_3_	100	>99
7.	**Ru‐1** (0.5 mol%)	KO*t*Bu (5%)	90	60	3	CHCl_3_	100	>99
8.	**Ru‐1** (0.5 mol%)	—	90	60	3	CHCl_3_	54^d^	47^d^
9.	**Ru‐1** (0.5 mol%)	KO*t*Bu (5%)	35	20	40	CHCl_3_	100	>99

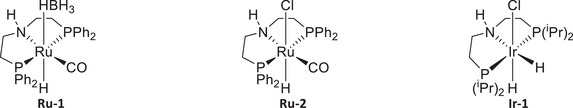

^a^ General reaction conditions: 1 mmol of NBR as starting material, 0.05 mmol of KO*t*Bu, 3 mL of THF, 1 mL of *i*PrOH, in 10 mL microwave vial sealed inside a 500 mL autoclave.

^b^ Conversion is estimated by the consumption of C**H**CN signal in ^1^H NMR spectrum (*δ* 2.6 ppm) and ^13^C NMR (δ 122 ppm).

^c^ Yield is based on the isolated weight and analysis by NMR and IR spectroscopy.

^d^ Calculated by integrating the amine signal at ∼1500 cm^−1^ in the IR spectrum. The conversion and yield in this case could not be estimated by NMR spectroscopy due to overlapping signals of C**H_2_** NH_2_ (*δ* 2.6 ppm) and C**H**CN (*δ* 2.6 ppm).

In a control experiment, no hydrogenation of polybutadiene (*M*_n_ = ∼5,000 g/mol) was observed under similar conditions (Figures S250 and S251). Interestingly, the IR spectrum (from entry 2, Table 1) showed the appearance of signals at 805 and 701 cm^−1^, suggestive of *cis*‐butadiene isomer, which were not present in the initial NBR that contained primarily the *trans* isomer. This is in line with previous reports confirming that analogous pincer catalysts can perform C═C isomerization [41, 42, 43]. Additionally, heating allyl cyanide in the presence of 1 mol% Ru‐MACHO BH (**Ru‐1**) catalyst in THF resulted in the formation of *cis*/*trans* crotononitrile (43% yield) in 3:4 ratio confirming the ability of Ru‐MACHO BH catalyst to perform isomerization (Figure S57). Remarkably, no secondary or tertiary amines were observed in the NMR spectra (from entry 2, Table 1), suggesting the reaction to be highly selective towards the formation of linear polyamines. Performing the reaction without Ru‐MACHO‐BH but in the presence of KO*t*Bu did not lead to any conversion of NBR, suggesting the crucial role of ruthenium in the hydrogenation process (Table 1, entry 3). Performing the hydrogenation reaction using Ru‐MACHO (**Ru‐2**) and KO*t*Bu (5%) also produced polyamine in quantitative yield (Table 1, entry 4). However, **Ir‐1** under the same condition produced an insoluble material which could not be fully characterized.

The hydrogenation reaction also exhibited a quantitative yield of polyamine in 3 h, albeit at 90°C using either 1 mol% (entry 6) or 0.5 mol% catalytic loading of **Ru‐1** and KO*t*Bu (5%) (entry 7, Table 1). Performing this reaction in the absence of base showed only 54% conversion of NBR, confirming the significance of KO*t*Bu under lower catalytic loading (entry 8). Notably, the hydrogenation of NBR to polyamine was also obtained in quantitative yield at 35°C when the reaction was run for 40 h (entry 9, Table 1). To the best of our knowledge, this represents the mildest temperature reported for the hydrogenation of nitriles to primary amines. Lowering the catalytic loading to 0.1 mol% did not lead to the full hydrogenation of NBR (Table S2, entries 10 and 11).

After successfully achieving the catalytic hydrogenation of NBR into polyamines, we paid attention to its transformation into polyols. Our strategy was inspired by reports on the transformation of small‐molecule nitriles to alcohols using catalytic hydrogenation in the presence of water [44, 45, 46]. We started our investigation by studying ruthenium and manganese catalysts previously reported for the hydrogenation of nitriles [44, 45, 46]. In a pilot experiment, 100 mg (0.94 mmol) of NBR was suspended in THF/H_2_O (1.5/0.5 mL) in a pressure reactor under argon in the presence of 1 mol% Ru‐MACHO‐BH complex (**Ru‐1**). The reactor was pressurised with 40 bar H_2_ and heated at 150°C for 20 h (Table 2, entry 1). Analysis of the isolated crude product by NMR and IR spectroscopies revealed 38% conversion of nitrile to alcohol (38% yield). In pursuit of more efficient catalysts, we studied other ruthenium catalysts reported for the transformation of nitriles to alcohols. **Ru‐2** (1 mol%) in the presence of KO*t*Bu (5 mol%) behaved similarly to that of **Ru‐1,** producing 37% yield of polyol (Table 2, entry 2). We hypothesized that acidic conditions might favor the hydrolysis of imines that would be needed for the conversion of nitriles to alcohols (*vide infra*, Figure 4) [47].

**TABLE 2:** Optimization of precatalyst choice for the hydrogenation of NBR to polyol.^a^ ^.^

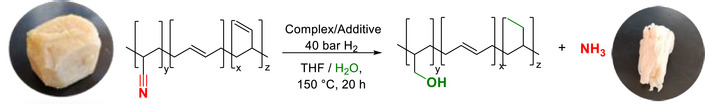						
Entry	Pre‐catalyst	Triphos (mol %)	Additive (mol %)	Solubility	Conversion (%)^b^	Yield (%)^c^
1.	**Ru‐1** (1 mol%)	—	—	DMSO, DMF	38	38^d^
2.	**Ru‐2** (1 mol%)	—	KO*t*Bu (5%)	DMSO, DMF	38	37^d^
3.	**Ru‐3** (1 mol%)	2%	PTSA (15%)	DMSO, DMF	100	>99
4.	**Ru‐4** (1 mol%)	—	—	Negligible	—	—
5.	**Ru‐5** (1 mol%)	—	—	Negligible	—	—
6.	—	—	PTSA (15%)	—	0	0
7.	**Ru‐3** (1 mol%)	—	PTSA (15%)	—	0	0
8.	—	2%	PTSA (15%)	—	0	0
9.^e^	**Ru‐3** (1 mol%)	2%	PTSA (15%)	Negligible	—	—

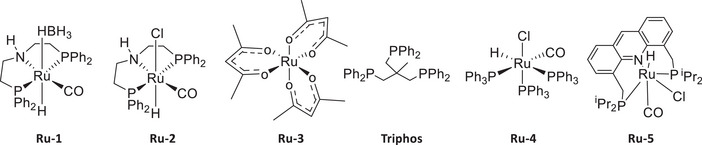

^a^ Reaction conditions: NBR (0.94 mmol/100 mg), THF (1.5 mL), H_2_O (0.5 mL), 150°C, 40 bar H_2_ added at room temp.

^b^ Conversion is estimated by the consumption of the C**H**CN signal in the ^1^H NMR spectrum (*δ* 2.8 ppm) and ^13^C NMR spectrum (*δ* 122 ppm).

^c^ Yield is based on the isolated weight and analysis of NMR and IR spectroscopy.

^d^ Yield is determined by ^1^H NMR spectroscopy by the integration of C**H_2_** OH signals.

^e^ Solvent: 2 mL THF.

We therefore considered using Ru/Triphos‐based systems that have been reported for the hydrogenation of polar groups such as esters, amides, acids, anhydrides, carbonates, urea, as well as polyesters, polycarbonates, and recently epoxy‐resins [10, 48, 49, 50]. Remarkably, the use of 1 mol% Ru(acac)_3_ (**Ru‐3**), Triphos (2 mol%), and 15 mol% PTSA (*p*‐toluene sulfonic acid) led to the complete conversion of nitrile groups to alcohols (Table 2, entry 3; Figure 2C–E). Interestingly, in this case, terminal C═C groups were found to be hydrogenated (exhibiting a new signal at *δ* 0.8 ppm in ^1^H NMR spectrum corresponding to CH_3_ protons, Figure 2C), whereas the internal ones remained unreacted. Like polyamine, the isomerisation of *trans* internal alkenes to *cis* was also observed in this case, as evidenced by the appearance of signals at 691 and 811 cm^−1^ in the IR spectrum (Figure 2E).

Complexes **Ru4** and **Ru5** under similar conditions but without using any additive also catalysed the hydrogenation process, forming a polyol but also led to the formation of an insoluble material indicative of a cross‐linked polymer as per the IR spectra (Table 2, entries 4 and 5) [51]. Control reactions using PTSA (without **Ru‐3** or Triphos, Table 2, entry 6), or **Ru‐3** (without Triphos, Table 2, entry 7), or Triphos and PTSA (without **Ru‐3**, Table 2, entry 8) did not lead to any conversion of NBR or the formation of polyol, suggesting the importance of all three components in catalytic transformation. Performing the reaction without water (Table 2, entry 9) produced an insoluble material which could not be properly characterised.

Studies described in Table 2 suggested Ru‐Triphos/PTSA to be the most efficient catalytic system for the transformation of NBR to polyol. Encouraged by this study, we conducted further optimisation to improve the catalytic turnover number. Lowering the reaction temperature to 100°C (Table 3, entry 2) from 150°C (Table 3, entry 1) stopped the reaction, showing no conversion of the NBR, confirming the need for a higher temperature. Using MSA (methane sulfonic acid) and Hf(OTf)_4_ (hafnium triflate) instead of PTSA also showed quantitative conversion of NBR to polyol in 20 h (Table 3, entries 3 and 4). However, reducing the reaction time to 6 h showed MSA to be a more effective acid than PTSA and Hf(OTf)_4_ (Table 3, entries 5–7). Under milder hydrogen pressure (20 bar), entries 8 and 9 demonstrate that the reduction of nitrile groups to alcohols remains efficient (> 99% yield) using **Ru‐3** (0.5 mol%), Triphos (1 mol%), and either PTSA or MSA (15 mol%) as additives. However, a key difference emerges in chemoselectivity: at 20 bar, the vinyl groups (1,2 CH═CH_2_) in the NBR backbone [52, 53] are not hydrogenated (Figures S103 and S106 – ^1^H NMR, *δ*_H_: 5.0 ppm). This suggests that while the system retains high activity for nitrile hydrogenation under reduced pressure, the hydrogenation of olefins is suppressed, allowing for the selective transformation of nitrile functionalities without compromising the polymer's unsaturated structure.

**TABLE 3:** Optimization of reaction conditions for the hydrogenation of NBR to polyols using **Ru‐3**.^a^ ^.^

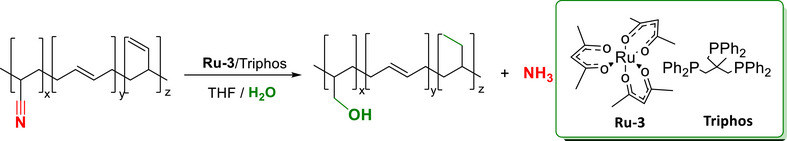						
Entry	Pre‐catalyst	Triphos (mol %)	Additive (mol %)	Time (h)	Conversion (%)^b^	Yield (%)^c^
1.	**Ru‐3** (0.5 mol%)	1%	PTSA (15%)	20	100	>99
2.^d^	**Ru‐3** (0.5 mol%)	1%	PTSA (15%)	20	0	0
3.	**Ru‐3** (0.5 mol%)	1%	MSA (15%)	20	100	>99
4.	**Ru‐3** (0.5 mol%)	1%	Hf(OTf)_4_ (15%)	20	100	>99
5.	**Ru‐3** (0.5 mol%)	1%	PTSA (15%)	6	8	7^e^
6.	**Ru‐3** (0.5 mol%)	1%	MSA (15%)	6	75	74^e^
7.	**Ru‐3** (0.5 mol%)	1%	Hf(OTf)_4_ (15%)	6	12	11^e^
8.^f^	**Ru‐3** (0.5 mol%)	1%	PTSA (15%)	20	100	>99
9.^f^	**Ru‐3** (0.5 mol%)	1%	MSA (15%)	20	100	>99
10.	**Ru‐3** (0.5 mol%)	1%	PTSA (5%)	20	0	—
11.	**Ru‐3** (0.5 mol%)	1%	MSA (1%)	20	100	>99
12.	**Ru‐3** (0.05 mol%)	0.1%	PTSA (15%)	20	100	>99
13.	**Ru‐3** (0.05 mol%)	0.1%	MSA (15%)	20	100	>99
14.	**Ru‐3** (0.05 mol%)	0.1%	MSA (5%)	20	48	47^e^
15.	**Ru‐3** (0.01 mol%)	0.02%	PTSA (15%)	20	2	—
16.	**Ru‐3** (0.01 mol%)	0.02%	MSA (15%)	20	43	42^e^
17.	**Ru‐3** (0.01 mol%)	0.02%	MSA (15%)	72	52	50^e^
18.^g^	**Ru‐3** (0.05 mol%)	0.1%	PTSA (15%)	68	100	>99

^a^ Reaction conditions: NBR (0.94 mmol or 100 mg), THF (1.5 mL), H_2_O (0.5 mL), 150°C.

^b^ Conversion is estimated by the consumption of the C**H**CN signal, estimated by ^1^H NMR (2.8 ppm) spectra taken in DMSO‐d6.

^c^ Yields are based on the weight of the isolated product.

^d^ 100°C.

^e^ Yield is determined by ^1^H NMR spectroscopy in DMSO‐d6 by the integration of C**H_2_** OH signals.

^f^ 20 bar.

^g^ 1 g NBR.

Such pressure‐dependent selectivity is particularly advantageous in fine‐tuning product properties for material applications. Reducing the loading of PTSA to 5 mol% shut down the catalysis (Table 3, entry 10), whereas in the case of MSA, the catalysis worked efficiently even with 1 mol% loading (Table 3, entry 11). Using 0.05 mol% of the **Ru‐3** precatalyst with either PTSA or MSA (15 mol%) results in quantitative conversion and yields after 20 h (Table 3, entries 12 and 13). A TON of 2000 observed here is the highest of previously reported analogous deaminative hydrogenation of small molecule nitriles to alcohols in peer‐reviewed literature [44, 45, 46]. However, a lower yield was obtained when using 5% MSA, suggesting the need for a higher amount of MSA (entry 14), as reported previously [54, 55]. Reducing the **Ru‐3** loading to 0.01 mol% led to a dramatic drop in performance, especially with PTSA, where conversion dropped to 2% (Table 3, entry 15). In contrast, MSA showed better performance at the same loading, achieving 48% conversion (Table 3, entry 16), suggesting it is a more effective additive. Increasing the reaction time to 72 h did not make any significant change in the conversion or yield (Table 3, entry 17). The process was also conducted on a gram scale using 0.05 mol% **Ru‐3,** which showed quantitative transformation of NBR to polyol (Table 3, entry 18).

Having in hand optimized methods for the hydrogenation of commercially available technical‐grade NBR, we studied the catalytic hydrogenation of post‐consumer NBR waste. It is worth noting that most NBR‐based consumer materials are vulcanized to enhance their mechanical strength, chemical resistance, and thermal stability, making them suitable for demanding applications such as gloves, seals, and gaskets [56, 57]. To enable efficient hydrogenation, the end‐of‐life NBR waste was first devulcanized to break the crosslinked network. Without devulcanization, the post‐consumer NBR plastics were not soluble even on prolong heating at 150°C. Nitrile gloves and O‐rings were devulcanized by refluxing them in nitrobenzene as per a method reported in the literature [58] producing a dark brown/black material soluble in THF and partially soluble in DMSO, which showed signals characteristic of NBR in NMR and IR spectra. The devulcanization step also enabled the recovery of butylated hydroxytoluene (BHT) as a yellow powder in ∼5% yield, a chemical commonly used as a stabiliser in NBR products.

Under previously optimized conditions for NBR — **Ru‐1** (1%) and KO*t*Bu (5%) at 60 bar H_2_, devulcanized materials obtained from gray gloves were hydrogenated to produce the corresponding polyamine in quantitative yield (Table 4, entry 1). Similarly, using the conditions optimised for polyols — **Ru‐3** (1%), PTSA (15%), and Triphos (2%) at 40 bar H_2_, gray gloves were transformed to the corresponding polyol in quantitative yield (Table 4, entry 2). A catalytic loading of 0.05% **Ru‐3** didn't lead to the full conversion of the NBR obtained from the gray gloves (Table 4, entry 3). Similarly, devulcanized O‐rings were successfully hydrogenated to the corresponding polyamine and polyol in a quantitative yield (Table 4, entries 4 and 5). Furthermore, a devulcanized sample of blue gloves was also transformed to polyol and polyamine in quantitative yield under our reaction conditions (Table 4, entries 6 and 7). These results indicate that both technical‐grade and end‐of‐life NBR materials respond similarly under our catalytic conditions and can be effectively converted into polyamines and polyols.

**TABLE 4:** Hydrogenation of post‐consumer NBR waste into polyamines and polyols.^a^

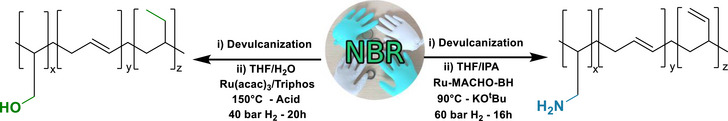						
Entry	NBR Source	Precatalyst (mol %)	Additive (mol %)	Polymer obtained	Conversion (%)^b^	Yield (%)^c^
1.	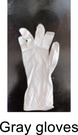	**Ru‐1** (1%)	KO*t*Bu (5%)	Polyamine	100	>99
2.		**Ru‐3** (1%)	PTSA (15%) Triphos (2%)	Polyol	100^d^	>99^d^
3.		**Ru‐3** (0.05%)	PTSA (15%) Triphos (0.1%)	Polyol	53^d^	52^d^
4.	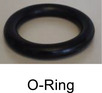	**Ru‐1** (1%)	KO*t*Bu (5%)	Polyamine	100	>99
5.		**Ru‐3** (1%)	PTSA (15%) Triphos (2%)	Polyol	100^d^	>99^d^
6.	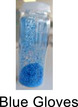	**Ru‐1** (1%)	KO*t*Bu (5%)	Polyamine	100	>99
7.		**Ru‐3** (1%)	PTSA (15%) Triphos (2%)	Polyol	100^d^	>99^d^

^a^ Hydrogenated products were soluble in DMSO, DMF, CHCl_3_, and CH_3_OH.

^b^ Conversion estimated by the consumption of C**H**CN signal in ^1^H NMR at 2.8 ppm (DMSO‐d6)/ 2.6 ppm (CDCl_3_).

^c^ Yield is based on isolated weight and characteristic NMR signals of polyol and polyamine.

^d^ Estimated based on the NMR spectrum analysis (see Supporting Information, Section 8).

**FIGURE 3:**
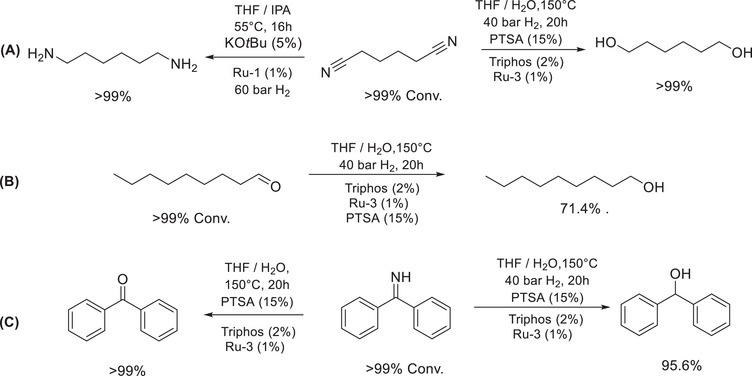
Control experiments for mechanistic studies. Hydrogenation of adiponitrile (A), nonanal (B), and benzophenone imine.

To confirm the reaction pathways of both the transformations described above, a series of control experiments was performed (Figure 3). Under the reaction conditions described in Table 1, entry 2 using Ru‐MACHO‐BH (**Ru‐1**, 1 mol%), and KO*t*Bu (5 mol%), adiponitrile was selectively hydrogenated to 1,2‐diaminohexane in a quantitative yield, confirming the catalyst's ability to hydrogenate small molecules or oligonitrile. Additionally, using **Ru‐3** (1 mol%), Triphos (2%), and PTSA (15%), adiponitrile was completely transformed to 1,6‐hexanediol selectively (Figure 3A). Furthermore, an aliphatic aldehyde, nonanal was hydrogenated to nonanol in high yield using **Ru‐3**/Triphos system, confirming the catalyst's ability to hydrogenate aldehydes to alcohols (Figure 3B). Similarly, a primary imine (benzophenone imine), was efficiently and selectively converted to the corresponding alcohol and ketone in the presence and absence of H_2_,O respectively (Figure 3C). Regarding polyols, the hydrogenation of NBR in the absence of both **Ru‐3**/Triphos and H_2_ but in the presence of PTSA and water resulted in no conversion of nitriles, excluding the possibility of acid‐catalysed hydrolysis of nitriles to amides or carboxylic acids (Supporting Information, S9, Figure S235). The reaction conducted in the presence of **Ru‐3**/Triphos, PTSA, and water but without molecular hydrogen also showed no conversion, demonstrating that hydrogen pressure is essential for this transformation (Figure S237). Notably, in the absence of H_2_O, the catalytic hydrogenation of NBR resulted in the formation of an insoluble rubbery material. Analysis of this material by IR spectroscopy showed the transformation of the nitrile moiety along with crosslinking of NBR through double bonds (Figure S81).

**FIGURE 4:**
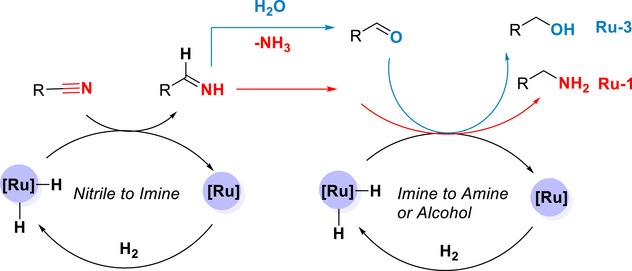
Proposed general pathway for the hydrogenation of nitriles to primary amines (in case of Ru‐MACHO‐BH, **Ru‐1** catalyst) or alcohols (in case of **Ru3**‐Triphos catalyst) [36, 44].

Based on these results and previous reports in the literature [36, 44, 45], we propose a reaction pathway as outlined in Figure 4. We suggest that first the nitrile moiety undergoes hydrogenation in the presence of ruthenium catalysts (either Ru‐MACHO‐BH, **Ru‐1**, or Ru/Triphos, **Ru‐3**) to produce an imine intermediate. This imine intermediate can undergo hydrogenation in the case of **Ru‐1** to form the corresponding primary amines. In the other case, when water is present, we suggest that imine undergoes hydrolysis to form an aldehyde intermediate that subsequently gets hydrogenated to an alcohol.

**FIGURE 5:**
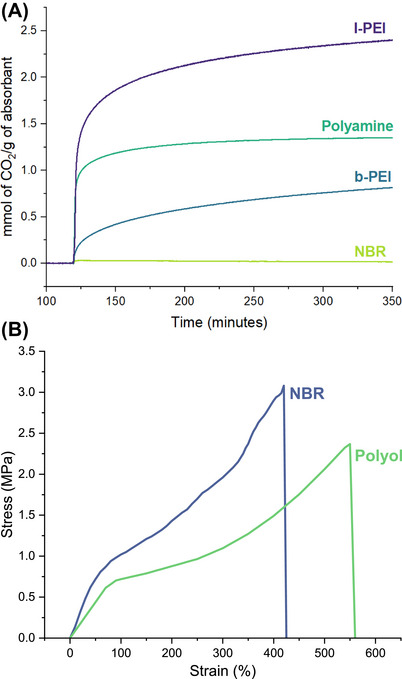
(A) CO_2_ capture over time for linear PEI (l‐PEI), polyamine (made from the hydrogenation of NBR), branched PEI (b‐PEI), and NBR. (B) Stress–strain plot for polyol and NBR samples.

Having developed catalytic processes for the synthesis of polyamines and polyols, we studied their properties for potential applications. Considering significant applications of polyamines in CO_2_ capture [59, 60, 61], we used the polyamine prepared in Table 1, entry 2 for CO_2_ capture using simultaneous thermal analysis (STA). The thermogravimetric analysis of this polymer showed the onset temperature (estimated as 10% wt loss) to be ∼ 440°C (Figure 2F). Polyamines were mixed with fumed silica in a 1:1 ratio to increase the porosity of the absorbent for efficient CO_2_ capture. The absorbent sample (67.65 mg) was first heated at 120°C for 100 min under nitrogen to allow elimination of any residual solvent and ensure no change in mass under a nitrogen atmosphere. The temperature was then dropped to 90°C, and the gas environment was changed from nitrogen to CO_2_ for the next 350 min. A sharp rise in the mass of absorbent was observed in the presence of CO_2,_ and over 350 min, the polyamine (produced using the method described in Table 1, entry 2) captured 59 mg (or 1.34 mmols) of CO_2_/g of absorbents (Figure 5A). In a control experiment, when NBR + fumed silica was subjected to the same study, only 0.71 mg (0.015 mmol) of CO_2_ was captured per gram of absorbent, confirming that indeed these polyamines are promising materials for CO_2_ capture. Additionally, a commercially available branched PEI (polyethylenimine, *M*_n_ = 10 000 g/mol), and a linear PEI (*M*_n_ = 2100 g/mol) captured 0.82 and 2.41 mmol of CO_2_/g of absorbent, respectively, under the same conditions. Higher CO_2_ capture in the case of linear PEI is likely due to a higher amine (primary and secondary) percentage by weight in linear PEI in comparison to polyamine made from NBR, which would have less amine percentage by weight due to the presence of butadiene copolymer.

To gain some understanding of the potential applications of the produced polyols, we studied their thermal and mechanical properties. Thermogravimetric analysis of the polymer obtained in Table 3, entry 1, showed the onset temperature to be ∼ 409°C, estimated as 10% weight loss (Figure 2F). Rectangular specimens of the polyol and NBR elastomers were characterised in tension using the strip‐test configuration described in ASTM D882 / ISO 1926. Remarkably, the synthesised polyol (produced using the method described in Table 3, entry 1) demonstrated greater ductility than the commercial NBR, reaching an elongation at break of ≈ 550% versus ≈ 420% for NBR (Figure 5B). Although its ultimate tensile strength was found to be lower (polyol 2.37 MPa vs. NBR 3.08 MPa), the polyol's superior stretchability highlights its potential where high extensibility is more critical than maximum load‐bearing capacity.

Finally, we also performed a preliminary life cycle assessment (LCA) by conducting cradle‐to‐gate footprint evaluation to estimate global warming potential (GWP), acidification, eutrophication, and water/energy consumption using the ACS Green Chemistry Institute's streamlined PMI‐LCA tool [62, 63]. A comparative hot‐spot analysis in a streamlined life cycle assessment approach showed improved environmental benefits for the hydrogenation approach in comparison to that using LiAlH_4,_ which is the only alternative method reported in peer‐reviewed literature for the transformation of NBRs to polyamines (Figures S256 and S257). Similarly, a significantly lower E‐factor was estimated for the transformation of post‐consumer NBR waste involving devulcanization and hydrogenation steps in comparison to that using LiAlH_4_.

## 3 Conclusion

In conclusion, we report here two new directions for the chemical recycling of post‐consumer NBR waste. In the first direction, NBR can be hydrogenated to make linear polyamines selectively using Ru‐MACHO‐BH catalyst under mild reaction conditions (e.g., 35°C–55°C). In the other approach, when water is used as a part of the solvent, NBR can be hydrogenated to make a linear polyol selectively. Ru/Triphos catalyst in the presence of a Bronsted acid such as PTSA and MSA was found to be the most effective for the formation of polyol, exhibiting a turnover number for ruthenium up to 2000. These processes were demonstrated for both technical‐grade NBRs as well as post‐consumer NBR waste sourced from nitrile gloves and O‐rings. A mechanism based on control experiments and previous reports has been proposed (Figures 3 and 4), suggesting that nitrile first gets hydrogenated to imines, followed by either its subsequent hydrogenation to amines (for polyamines) or deaminative hydrolysis to make aldehydes that get hydrogenated to make alcohols (for polyols). Finally, polyamines were demonstrated to be a promising material in CO_2_ capture, whereas the polyol's mechanical properties are suggestive of their potential use in stretch‐demanding applications. These studies open new possibilities for upcycling NBRs to make potentially high‐value materials.

## Conflicts of Interest

The authors declare no conflicts of interest.

## Supporting information




**Supporting File 1**: Supporting information contains experimental details related to catalytic and mechanistic studies, characterization of polymers, as well as details on CO_2_ capture and mechanical properties.

## Data Availability

The data that support the findings of this study are available in the supplementary material of this article. The raw research data supporting this publication can be accessed at https://doi.org/10.17630/1055803e‐69dd‐42f1‐a3e0‐d0fdd75983a1.
