# Supplementary material for: Chemical Upcycling of Nitrile Butadiene Rubbers to Polyamines and Polyols by Chemoselective Catalytic Hydrogenation
Source: Angew Chem Int Ed Engl. 2026 Mar 18;65(18):e25705. doi: 10.1002/anie.202525705 (PMC13110760; doi:10.1002/anie.202525705)
Supplement: Supplementary file 1 — Supporting File 1: Supporting information contains experimental details related to catalytic and mechanistic studies, characterization of polymers, as well as details on CO2 capture and mechanical properties. [file ANIE-65-e25705-s001.docx]

**Supplementary information**

**Chemical Upcycling of Nitrile Butadiene Rubbers to Polyamines and Polyols by Chemoselective Catalytic Hydrogenation**

Alejandra Sophia Lozano Perez^[a]^, Raymundo Marcial-Hernandez^[a]^, Harini Sampathkumar,^[b]^ Oluchi Emenike,^[b]^ Ketan Pancholi,^[b]^ Claire N. Brodie,^[a]^ Daniel M. Dawson^[a]^ and Amit Kumar*^[a]^

[a] EaStCHEM, School of Chemistry, University of St Andrews

North Haugh, KY16 9ST St Andrews, (UK)

[b] School of Computing, Engineering and Technology, The Sir Ian Wood Building, Robert Gordon University, Garthdee Rd, Garthdee, Aberdeen AB10 7GE, (UK)

E-mail: ak336@st-andrews.ac.uk

Table of Contents

[**S1 General information** 3](#_Toc220880748)

[**S1.1 General procedure for the hydrogenation of nitriles into amines** 4](#_Toc220880749)

[**S1.2 General procedure for the hydrogenation of nitriles to alcohols** 5](#_Toc220880750)

[**S2 Characterization of Starting Material – NBR** 5](#_Toc220880751)

[**S3 Method to calculate the conversion of nitrile** 10](#_Toc220880752)

[**S4 Types of crosslinking observed** 13](#_Toc220880753)

[**S5 Full NMR Characterization of the Polyamine** 14](#_Toc220880754)

[**S6 Full NMR Characterization of the polyol** 42](#_Toc220880755)

[**S7 Kinetic studies of Nitrile consumption into alcohol** 91](#_Toc220880756)

[**S8 Catalytic hydrogenation of NBR end-of-life products** 98](#_Toc220880757)

[*Starting Material characterization* 98](#_Toc220880758)

[*Devulcanization procedure* 105](#_Toc220880759)

[*End-of-life materials post-hydrogenation* 120](#_Toc220880760)

[**S9 Small molecule experiments** 143](#_Toc220880761)

[**S10 CO_2_ Capture tests** 154](#_Toc220880762)

[**S11 Mechanical properties** 158](#_Toc220880763)

[**S12 Economic and environmental assessment** 158](#_Toc220880764)

[S12.1 Life cycle assessment: 158](#_Toc220880765)

[S12.2 E Factor 160](#_Toc220880766)

[**S13 References** 160](#_Toc220880767)

# **S1 General information**

Unless otherwise specified, all manipulations were carried out under an argon atmosphere using standard Schlenk line techniques. All glassware was oven-dried and cycled under vacuum prior to use. Tetrahydrofuran (THF) was dried using a Grubbs-type solvent purification system (Innovative Technologies SPS) equipped with a degassing unit and subsequently stored over activated 4 Å molecular sieves. Deionized water was degassed before each use.

Poly(acrylonitrile-co-butadiene) (NBR) was bought from Sigma Aldrich with a 37-39 wt. % of acrylonitrile (Product No.180912). Ruthenium(III) acetylacetonate and 1,1,1 tris(diphenylphosphinomethyl)ethane were purchased from Fluorochem (Product F996216) and Sigma-Aldrich (Product No. 380741), respectively. Chlorocarbonylhydrido[4,5-bis-(di-i-propylphosphinomethyl)acridine]ruthenium(II), Milstein Acridine Catalyst (Strem - Catalog Number. 44-0525), Carbonylchlorohydridotris(triphenylphosphine)ruthenium(II) (ThermoScientific - Catalog number. 044955.03), Ru-Macho-BH (TCI- Product Number. R0137) were purchased from commercial suppliers as mentioned in parentheses. Mn-Macho-IPr was synthesised following the procedure described in the literature.^[1]^ Polyethyleneimine branched M_n_ = 10,000 Da was purchased from ThermoFisher Scientific (Catalog number. 040528.22). Polyethylenimine, linear (M_n_ = 2,100), and Polybutadiene (M_n_ = 5000) were purchased from Sigma-Aldrich (Product No. 764604, 383694). Nitrile gloves were purchased from Fisher scientific (Kimtech™ Sterling™ Nitrile Ambidextrous Gloves. Product Code. 10634525), O ring was purchased from CUPAR BEARINGS & TRANS LTD (Cupar, Scotland).

All catalytic reactions were carried out in 500 mL autoclaves. Analytics: ^1^H, ^13^C NMR data were recorded on a Bruker AVIII HD 500 spectrometer using DMSO-d6, and CDCl_3_ solvents. GC-MS data were collected on an Agilent 8860 GC system coupled to an Agilent 5977B EI instrument. The samples were prepared using an HPLC-grade DCM solvent. Infrared spectra (ATR-FTIR) were collected using a Shimadzu IRAffinity-1. TGA was performed using a Stanton Redcroft STA-780 Series Thermal Analyser between 20–600°C at a heating rate of 10 °C/min under a flow of nitrogen gas (30 mL/min). For CO_2_ capture tests, the NETZSCH STA49 F5 thermogravimeter was used under a flow of nitrogen and CO_2_. Gel permeation chromatography (GPC) was performed on an Agilent 1260 InfinityLab II GPC fitted with a refractive index (RI) detector (35 °C). The single (plus guard column) Agilent PolarGel column setup was contained within an oven (55 °C). DMF was used as the eluent at a flow rate of 1.0 mL min^−1^. Samples were dissolved in the eluent (2.0 mg mL^−1^), filtered (0.2 μm pore size) and run immediately. The calibration was conducted using a series of monodisperse polystyrene (M_n_ = 472 to 2’811.000 Da).

Solid-state ^13^C cross polarisation (CP) Magic Angle Spinning (MAS) NMR spectra were recorded using a Bruker Avance III equipped with a 9.4 T wide-bore superconducting magnet (^1^H and ^13^C Larmor frequencies of, respectively, 400.1 and 100.6 MHz) or a Bruker Avance Neo-X equipped with a 14.1 T wide-bore superconducting magnet (^1^H and ^13^C Larmor frequencies of, respectively, 600.1 and 150.9 MHz). Shredded glove samples were packed into standard Bruker 4 mm zirconia rotors. For the O-ring sample, pieces of chopped rubber were packed into the rotor with finely ground NaCl to provide stability when rotating the sample. The devulcanised materials were packed into standard Bruker 3.2 mm zirconia rotors. All samples were rotated at the magic angle at a rate of 12.5 kHz. The signal was enhanced using CP from ^1^H with a contact pulse (ramped for ^1^H) of 1.0-1.5 ms and high-power (ν_1_ ≈ 100 kHz) SPINAL-64 decoupling of ^1^H was applied during acquisition. Signal averaging was carried out for 2560, 3072, 17848, 30000 or 32768 transients (for blue, gray, O-ring, devulcanised O-ring and devulcanised glove samples, respectively) with a recycle interval of 3 s. Chemical shifts are reported in ppm relative to (CH_3_)_4_Si using L-alanine (δ(**C**H_3_) = 20.5 ppm) as a secondary solid reference.

Differential scanning calorimetry (DSC) measurements were performed using a DSC 204 Phoenix instrument equipped with a CC200 F1 controller (NETZSCH). Samples were analyzed in sealed aluminum crucibles under a nitrogen atmosphere with a flow rate of 40 mL min⁻¹. The temperature program consisted of an initial heating ramp from ambient temperature to 100 °C at a rate of 10 °C min⁻¹, followed by cooling to −80 °C and a subsequent second heating to 100 °C at the same rate. This heat–cool–heat protocol was applied to remove thermal history effects and ensure reproducible thermal transitions.

Residual ruthenium analysis was performed using 4210 Microwave plasma atomic emission spectrometers (MP-AES) from Agilent technologies. A stock solution of 3% HNO_3_ was used for preparing samples. The calibration curve was prepared by diluting a 1000 ppm Ru standard solution into 10, 20, 50 and 100 ppm solutions. The calibration curve and the samples were analysed three times at the characteristic’s wavelengths for Ruthenium (372.803, 349.894, 379.935 & 372.693), see below for the calibration example at 372.803 nm). The samples were prepared by dispersing the product (samples of devulcanized gray glove, Polyol from gray glove & polyamine from gray glove) into the HNO_3_ stock solution to generate a 0.1 mg/mL sample solution. Due to the low solubility of the material, all samples were heated at 70 °C under vigorous stirring overnight prior to the corresponding analysis. The samples were filtered and tested directly without further dilution.


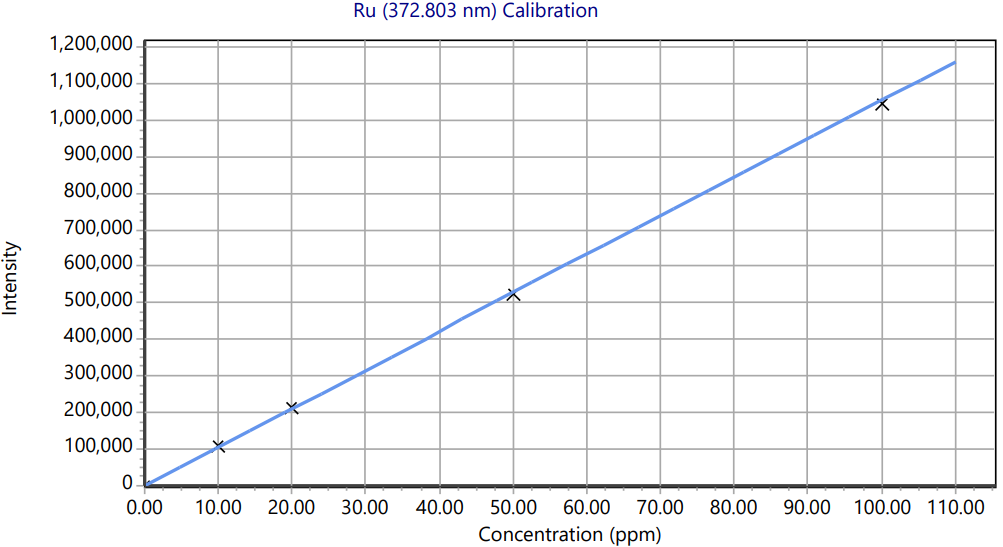


Figure: Calibration Standard for Ru at 372.803 nm.

(Y axis) Intensity= 10542.83 *Concentration+0.38701095

Correlation coefficient: 0.99999

Elemental CHN analysis was performed using a Thermo Fisher Scientific Flash SMART 2000 (Serial No. 2018.FLS0037). Samples were combusted in a CN/CHN prepacked quartz reaction tube (Elemtex, PXR1000) and separated using a 2 m stainless steel CN/CHN separation column (Elemental Microanalysis, E3023, Lot BN293467). The furnace temperature was set to 950 °C and the oven temperature to 60 °C. Helium was used as both carrier and reference gas at a flow rate of 140 mL min⁻¹, while oxygen was introduced at 250 mL min⁻¹ to ensure complete combustion. The total run time was 660 s, with a 12 s sampling delay and oxygen injection terminated after 5 s. Detection was carried out using a thermal conductivity detector (TCD).

Oxygen analysis was performed using a Thermo Fisher Scientific Flash SMART 2000 (Serial No. 2018.FLS0037). Samples were introduced into an O prepacked quartz pyrolysis tube (Elemtex, PP1000) and separated using a 1 m stainless steel O separation column (Thermo, Part No. 26007900, Lot BN292786). The furnace temperature was maintained at 1060 °C and the oven temperature at 65 °C. Helium was used as both carrier and reference gas at a flow rate of 100 mL min⁻¹. No oxygen flow was applied during the analysis. The total run time was 400 s with no sampling delay and no oxygen injection. Detection was carried out using a thermal conductivity detector (TCD) with a detector gain setting of 1.

Elemental CHNS analysis was carried out using a Thermo Fisher Scientific Flash SMART 2000 (Serial No. 2018.FLS0037). Samples were combusted in a CHNS prepacked quartz reaction tube (Elemtex, PXR1003) and separated using a 2 m PTFE CHNS separation column (CE Instruments, E3038, Lot BN312151). The furnace temperature was set to 950 °C and the oven temperature to 65 °C. Helium was used as the carrier gas at 140 mL min⁻¹ and as the reference gas at 100 mL min⁻¹, while oxygen was supplied at 250 mL min⁻¹ to ensure complete combustion. The total run time was 660 s, with a 12 s sampling delay and oxygen injection terminated after 5 s. Detection was performed using a thermal conductivity detector (TCD).

## **S1.1 General procedure for the hydrogenation of nitriles into amines**

In a dry 8 mL microwave vial, 0.9 mmol of polyacrylonitrile-co-butadiene rubber (100 mg) was added. The vial was sealed and purged under argon. THF (3 mL) was added to the purged vial to dissolve the polymer under vigorous stirring. In another vial, the corresponding catalyst (e.g. ruthenium Macho-BH, 1 mol%) and a base (e.g. potassium *tert*butoxide, 5 mol% (5.6 mg)) were added. The vial was sealed and purged under inert conditions, followed by the addition of dry isopropanol (1 mL) and vigorous stirring.

The catalyst solution was added to the polymer solution via syringe under argon. The lid of the vial containing the mixture solution was pierced with two needle tips to enable the hydrogenation process. Finally, the vial was transferred to a stainless-steel autoclave previously purged under argon and loaded with metal beads to facilitate uniform heating. The autoclave was sealed under argon, and then further purged with hydrogen (10 bar) and pressurized at the corresponding pressure (e.g. 50 bar). The autoclave was then placed on a preheated oil bath at the desired temperature (e.g. 55 °C). The reaction mixture was vigorously stirred for 2-40 h at 860 rpm.

Once the experiment was completed, the autoclave was allowed to cool down to room temperature in air, and then hydrogen was carefully vented until reaching atmospheric pressure. The solvent was removed under reduced pressure using a rotary evaporator and further dried in a vacuum oven at 50 °C.

## **S1.2 General procedure for the hydrogenation of nitriles to alcohols**

A magnetic stirring bar and 100 mg of NBR (0.9 mmol) were placed in a microwave vial and subjected to three vacuum-argon cycles using a Schlenk line. Subsequently, 1.5 mL of THF and 0.5 mL of deionized water were added to the vial, and the mixture was stirred for 10 minutes. The NBR remained insoluble in the solvent mixture.

In a separate microwave vial, both 0.019 g of Ru(acac)₃ and 0.0659 g of Triphos were weighed, purged with argon, and dissolved in 1 mL of THF to yield 0.05 M and 0.01 M stock solutions, respectively. A 1 M solution of acid e.g. p-toluenesulfonic acid (PTSA) was also prepared in THF.

To the NBR suspension, 0.15 mL of the acid e.g. PTSA solution (15 mol%), and 0.18 mL of the catalyst solution (1 mol % Ru(acac)_3_ and 2 mol% Triphos) were added, and the mixture was stirred for 5 minutes. The vial cap was then pierced with needles to allow hydrogen access, and the vial was placed inside a 500 mL autoclave under an inert argon atmosphere. Metal beads were added to promote efficient heat transfer. The autoclave was sealed, pressurized with 40 bar of H₂, and heated to 150 °C in an oil bath with stirring at 860 rpm for 20 hours.

After completion of the reaction time, the reaction mixture was found to be homogeneous in nature. The contents were concentrated under reduced pressure to remove the solvent and precipitate the polymer, which was subsequently washed with methanol to eliminate residual PTSA. The solid was dried overnight in a vacuum oven at 50 °C. The resulting material was analyzed by NMR spectroscopy, FT-IR, and thermogravimetric analysis (TGA).

# **S2 Characterization of Starting Material – NBR**

**^1^H NMR** (CDCl_3_, 500 MHz): δ_H_ 5.55-5.30 (HC=CH), 5.04 (H_2_C=CH), 2.78 (CHC≡N), 2.26 (H_2_CH=CH), 2.04 (CHCHC=CH_2_)_,_ 1.59 (CH_2_CHC≡N),1.24, 1.02 (CH_2_CH_3_)

**^13^C{^1^H} NMR** (CDCl_3_, 126 MHz): δ_c_ 134.77-125.29 (HC=CH), 122.4 (CN), 35.34, 30.34, (CH_2_CH=CH), 32.74 (CHCH_2_), 30.0 (CHCN and CH_2_CHCN)

**IR** (ATR-FTIR, cm^–1^): ν 2929m (C-H stretch), 2237sh (C≡N), 1635 (C=C stretch), 1436m(C-C), 968sh (CH bend from internal [1,4 trans] olefin), 916s (CH bend from vinyl olefin).


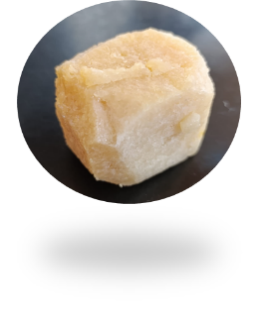


**d,g**

**h**

**c f**

**f f**

**b**

**a**

N

x

y

z

**a**

**b**

**c**

**d**

**d**

**c**

**e**

**f**

**g**

**h**

Figure S 1**.** ^1^H NMR spectrum (DMSO-d6, 500 MHz, 298K) of commercial Nitrile Butadiene Rubber.

**
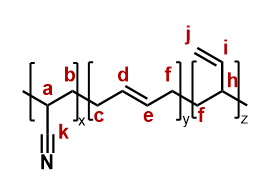
**

**f**

**f**

**c**

**d e**

**b**

**a**

**k**

Figure S 2. ^13^C{^1^H} NMR (126 MHz, DMSO-d6, 298K) spectrum of commercial Nitrile Butadiene Rubber.

Figure S 3. ^1^H, ^13^C- HSQC NMR (DMSO-d6) spectrum of commercial Nitrile Butadiene Rubber.

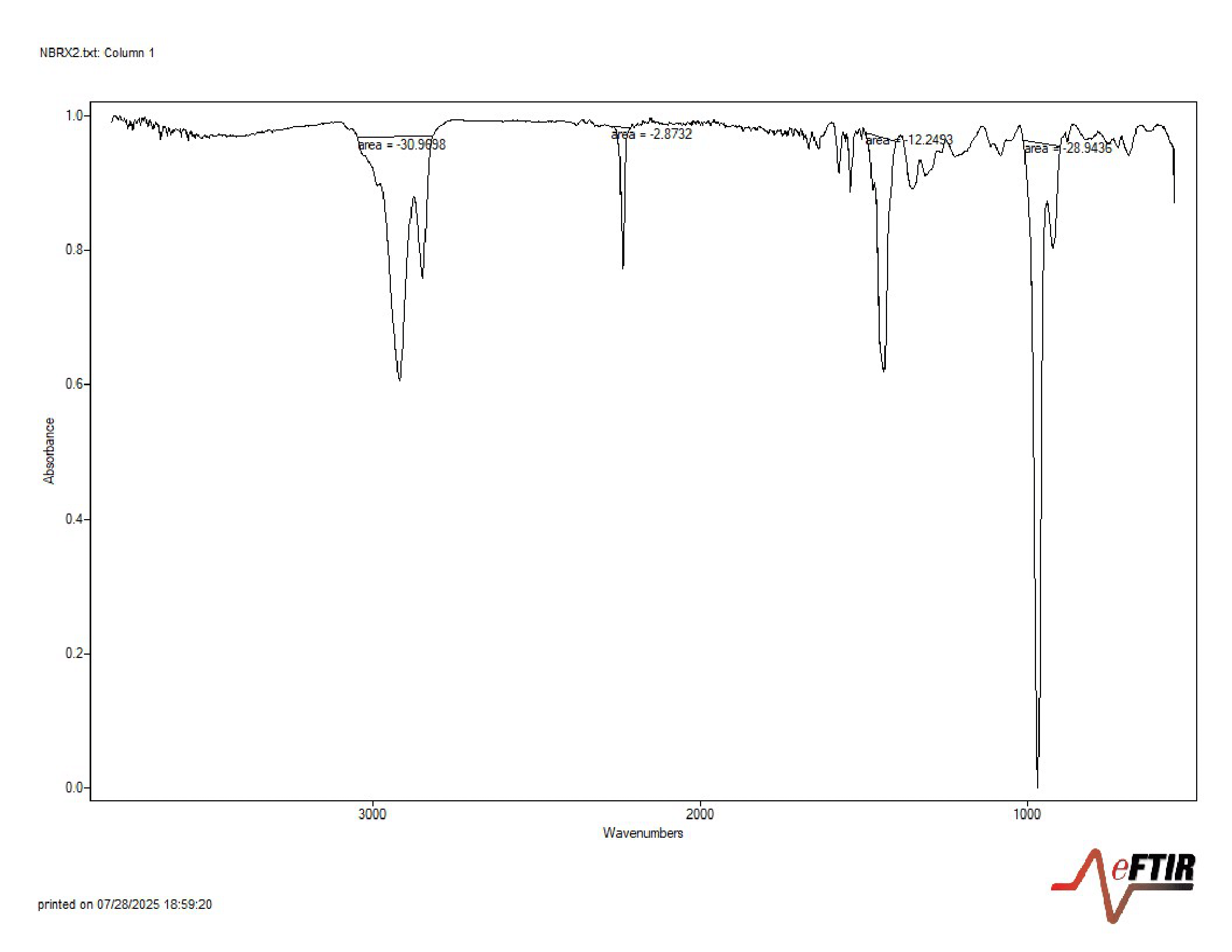


Figure S 4. (Top) ATR-FT-IR spectrum of commercial Nitrile Butadiene Rubber. (Bottom) ATR-FT-IR spectrum of commercial Nitrile Butadiene Rubber showing integration of signals.

Figure S 5. (a) TGA thermogram of commercial nitrile butadiene rubber sample with Onset temperature (10% wt loss.) of 404°C.

Figure S 6. GPC data of commercial NBR. (1) M_n_: 2101484 g/mol, PDI: 1.433 (2) M_n_: 27308 g/mol, PDI: 3.399..

**2**

**1**

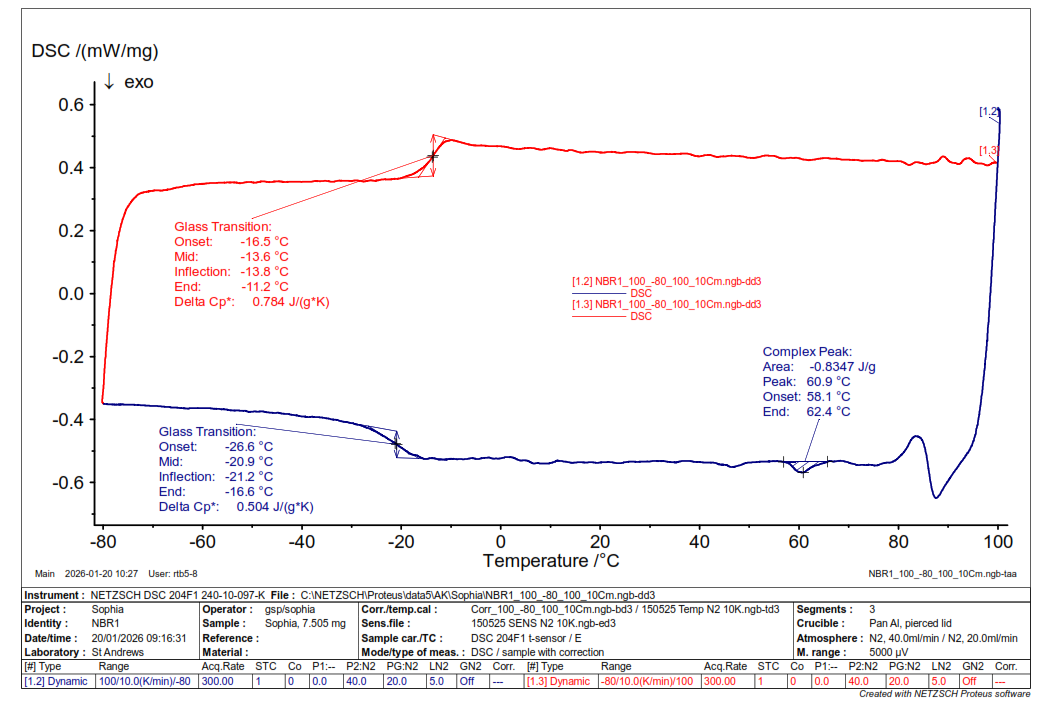


Figure S 7. DSC of commercial NBR showing glass transition temperature of -16.5°C.

Table S 1 Elemental analysis of commercial NBR.

C,79.39; H,8.65; N, 10.30; O, 1.32

# **S3 Method to calculate the conversion of nitrile**

The reaction mixture upon completion of the reaction time looked like a viscous heterogeneous material. The solvent was removed under vacuum, and the obtained residue was thoroughly washed with methanol and hexane and dried under vacuum (in a vacuum oven at 50 ^o^C) overnight to obtain a rubbery material. The obtained sample was weighed to estimate the yield and analysed by NMR and IR spectroscopy for characterisation.

In most cases, nitrile signals were found to have completely disappeared in ^1^H, HSQC, and ^13^C NMR spectra as well as IR spectra, confirming 100% conversion of nitrile. In a handful of cases, signals corresponding to nitrile were still present in the product mixture. The conversions in these cases were estimated by integrating the nitrile signal with respect to the internal HC=CH using IR spectroscopy (for polyamines, due to overlapping signals in ^1^H NMR spectroscopy), and ^1^H NMR spectroscopy (for polyols). The method involving an internal standard was not used as the product mixture was not homogeneous and needed prolonged stirring (a few hours to few days) for full solubility. Our current estimation could only work if the internal alkenes were not hydrogenated under our reaction conditions. To verify this hypothesis, we estimated the amount of internal HC=CH protons relative to mesitylene as an internal standard in NBR and a polyol produced from the hydrogenation of NBR. For this, a solution of 10 mg of NBR or polyol (obtained from the hydrogenation of NBR) was fully dissolved in 0.9 mL of DMSO to which a known amount of mesitylene was added. The amount of internal HC=CH protons was estimated as follows:

$$Mmol of internal double bond in NBR= \frac{\int double bond CH-{0.04}^{a}}{\int Mesitylene CH}*\frac{3 ( from no of protons in mesitylene)}{2 ( from no of protons in double bonds)}*mmol of mesityelene$$

$$Mmol of internal double bonds in NBR= \frac{0.749}{1.002}*\frac{3}{2}*0.074 mmol$$

$$Mmol of internal double bonds in NBR=0.083 mmol$$

Double bond

CH

*Mesitylene*

*(0.074 mmol)*

*3H*

Figure S 8. ^1^H NMR spectrum (DMSO-d6, 500 MHz, 298K) of commercial Nitrile Butadiene Rubber with internal standard mesitylene. ^a^ 0.04 comes from the signal of terminal 1,2 CH=CH_2_ present underneath the internal 1,4 C=C calculated by knowing the integration of 1,2 CH=CH_2_ (0.08). 0.749 comes from subtracting 0.04 from 0.789.

Similarly, the amount of HC=CH in polyamine and polyol was estimated as follows:

$$Mmol of internal double bond in Polyamine= \frac{\int double bond CH}{\int Mesitylene CH}*\frac{3 (comes from no of protons in mesitylene)}{2 (comes from no of protons in double bonds)}*mmol of mesitylene$$

$$Mmol of internal double bond in Polyamine= \frac{0.67}{1}*\frac{3}{2}*0.082 mmol$$

$$Mmol of internal double bond in Polyamine=0.083 mmol$$

$$Mmol of vinyl double bond in Polyamine=0.008 mmol$$

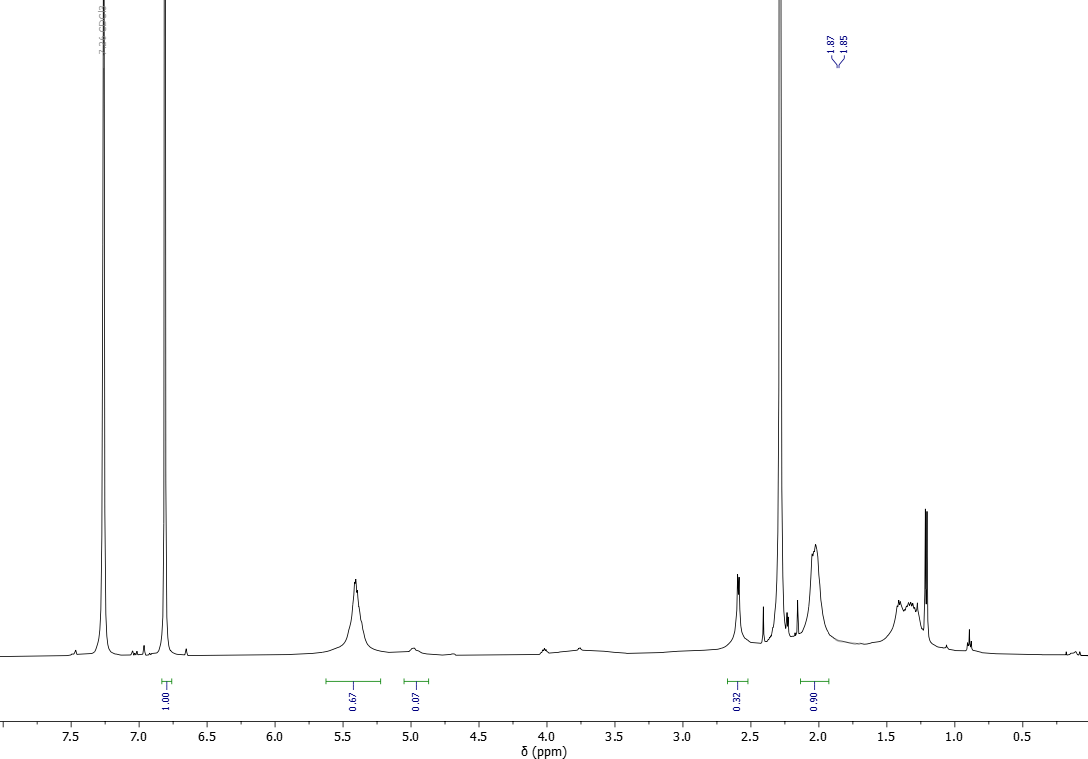


Double bond

CH

Mesitylene

0.082 mmol

3H

Figure S 9. ^1^H NMR spectrum (CDCl_3_, 500 MHz, 298K) of polyamine obtained from hydrogenation of Nitrile Butadiene Rubber (OH-NBR) with internal standard mesitylene.

$$Mmol of internal double bond in Polyol= \frac{\int double bond CH}{\int Mesitylene CH}*\frac{3 (comes from no of protons in mesitylene)}{2 (comes from no of protons in double bonds)}*mmol of mesityelene$$

$$Mmol of double bond in Polyol= \frac{0.672}{1.000}*\frac{3}{2}*0.080 mmol$$

$$Mmol of internal double bond in Polyol=0.081 mmol$$

Double bond

CH

*Mesitylene*

*(0.080 mmol)*

*3H*

Figure S 10. ^1^H NMR spectrum (DMSO-d6, 500 MHz, 298K) of polyol obtained from the hydrogenation of Nitrile Butadiene Rubber (OH-NBR) with internal standard mesitylene.

This confirmed that the HC=CH signal remains constant during hydrogenation and can be used as a reference to estimate the conversion, where full conversion was not observed. As described in Figure S1, the ratio of HC=CH (δ 5.48 ppm) to C**H**CN (δ 2.81ppm) was 2.750 to 1, hence, we set the double bond as the reference peak (δ 5.48 ppm) and calculated the conversion of the nitrile based on the change in integration of the signal at δ 2.81ppm (C**H**CN) relative to that of HC=CH (δ 5.48 ppm).

# **S4 Types of crosslinking observed**

Figure S 11. (a) Structure of NBR self-crosslinking through the double bond^[2]^ (b) Structure of Polyacrylonitrile self-crosslinking and forming aromatic rings.^[3]^ (c) Structure of NBR crosslinking through ketenimine.^[4]^

# **S5 Full NMR Characterization of the Polyamine**

Table S 2. Hydrogenation of NBR into polyamines^a^

*Table S2-Entry 1*

**^1^H NMR** (CDCl_3_, 500 MHz): δ_H_ 5.39 (HC=CH), 4.97 (HC=CH), 2.57 (H_2_CNH_2_), 2.03 (H_2_CH=CH), 1.41 (CHCH_2_NH_2_ or CHCH=CH_2_), 1.32 (CHCH_2_), 0.88 (CH_3_).

**^13^C{^1^H} NMR** (CDCl_3_, 126 MHz): δ_c_ 132.1-128.1 (HC=CH), 44.6 (CH_2_-NH_2_), 40.6 (CHCH_2_NH_2_), 34.6, 32.8, 30.0 (CH_2_CH=CH), 31.3 (CHCH_2_), 14.1 (CH_3_).

**IR** (ATR-FTIR, cm^–1^): ν 3352 (N-H stretch), 2915m (C-H stretch), 2235s (C≡N), 1560S (N-H bend, C=C stretch), 1438s (C-C), 966sh (HC bend from internal [1,4-trans] olefin), 914s (HC bend from 1,2 vinyl olefin).

Figure S 12. ATR-FT-IR spectrum of the polyamine produced from the hydrogenation of NBR. Table S2-Entry 1.


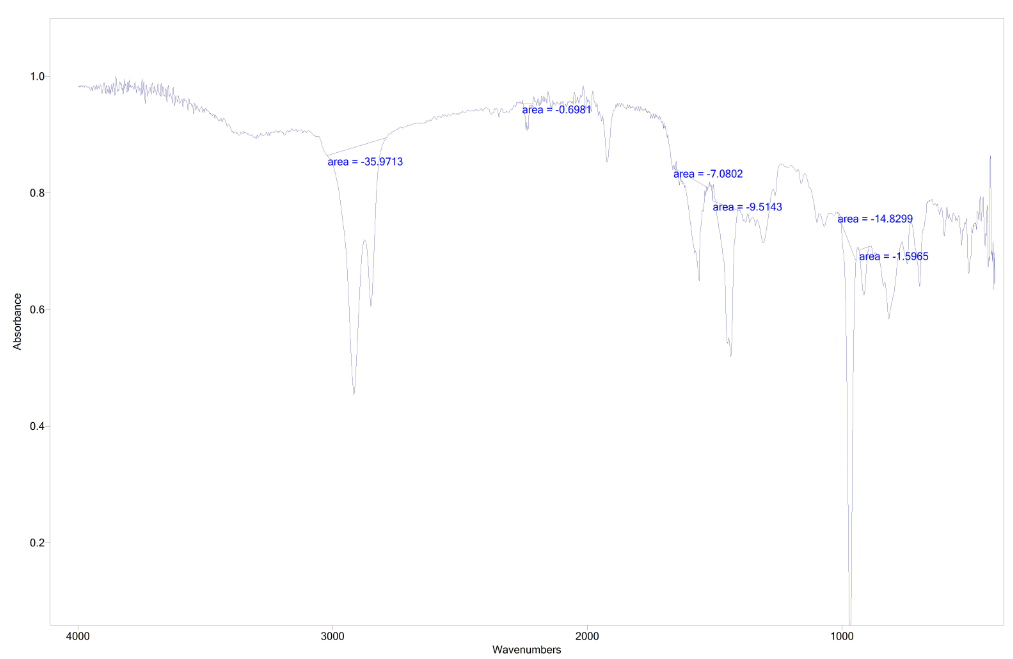


Figure S 13. ATR-FT-IR spectrum (integrated) of the polyamine produced from the hydrogenation of NBR. Table S2-Entry 1.

*
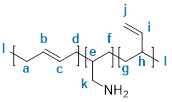
*

**b**

**c**

**i**

**l**

**e**

**h f**

**e f**

**a**

**d**

**j**

**k**

Figure S 14. ^1^H NMR spectrum (CDCl_3_, 500 MHz, 298K) of the polyamine produced from the hydrogenation of NBR. Table S2-Entry 1.

*
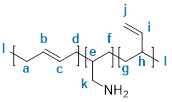
*

**k**

Figure S 15. ^13^C{^1^H} NMR (126 MHz, CDCl_3_, 298K) of the resulting product from hydrogenation of NBR to polyamine. Table S2-Entry 1.

*
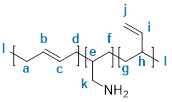
*

**k**

Figure S 16. ^1^H, ^13^C- HSQC NMR (CDCl_3_) spectrum of the polyamine produced from the hydrogenation of NBR. Table S2-Entry 1.

*Table S2-Entry 2*

**^1^H NMR** (CDCl_3_, 500 MHz): δ_H_ 5.43 (HC=CH), 4.99 (HC=CH), 2.61 (H_2_CNH_2_), 2.04 (H_2_CH=CH), 1.42 (CHCH_2_NH_2_ or CHCH=CH_2_), 1.35 (CHCH_2_).

**^13^C{^1^H} NMR** (CDCl_3_, 126 MHz): δ_c_ 132.0-128.1 (HC=CH), 44.7 (CH_2_-NH_2_), 40.6 (CHCH_2_NH_2_), 34.7, 32.8. 30.0 (CH_2_CH=CH), 31.3 (CHCH_2_).

**IR** (ATR-FTIR, cm^–1^): ν 3300 and 3352 (N-H stretch), 2915m (C-H stretch), 2235s (C≡N), 1562s (N-H bend, C=C stretch), 1433s (C-C), 968sh (HC bend from internal [1,4-trans] olefin), 912s (HC bend from 1,2 vinyl olefin).

*
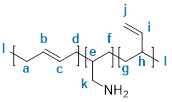
*

**b**

**c**

**i**

**e**

**h f**

**l**

**a**

**d**

**k**

**j**

Figure S 17. ^1^H NMR spectrum (CDCl_3_, 500 MHz, 298K) of the polyamine produced from the hydrogenation of NBR. Table S2-Entry 2.

*
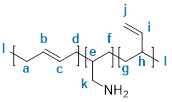
*

**k**

Figure S 18. ^13^C{^1^H} NMR (126 MHz, CDCl_3_, 298K) of resulting product from hydrogenation of NBR to polyamine. Table S2-Entry 2.

Figure S 19. ATR-FT-IR spectrum of the polyamine produced from the hydrogenation of NBR. Table S2-Entry 2.

Figure S 20. TGA thermogram of the polyamine produced from the hydrogenation of NBR. Table S2– Entry 2. Decomposition temperature: 439.1 °C calculated at 10% mass loss.


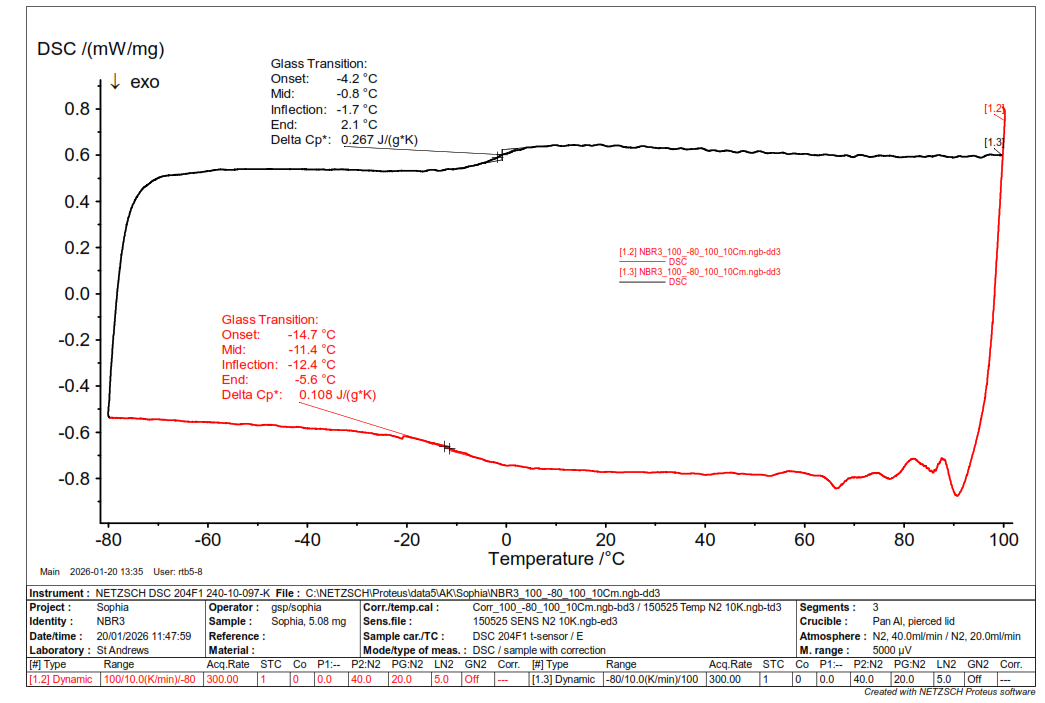


Figure S 21. DSC of polyamine produced from the hydrogenation of NBR. Amorphous material with glass transition of -4.2°C.

Table S 3 Elemental analysis of polyamine produced from the hydrogenation of NBR.

C,76.66; H,8.91; N, 8.62

*Table S2-Entry 3.*

**^1^H NMR** (CDCl_3_, 500 MHz): δ_H_ 5.54 (HC=CH), 5.40 (HC=CH), 2.60 (C≡N), 2.28 (H_2_CH=CH), 2.09 (CHCHC=CH_2_)_,_ 1.66 (CH_2_CHC≡N),1.31.

**IR** (ATR-FTIR, cm^–1^): ν 2918m (C-H stretch), 2235sh (C≡N), 1560 (C=C stretch), 1440m(C-C), 968sh (HC bend from internal [1,4-trans] olefin), 921s (HC bend from 1,2 vinyl olefin).

**a**

**h**

Figure S 22. ^1^H NMR spectrum (CDCl_3_, 500 MHz, 298K) of the polyamine produced from the hydrogenation of NBR. Table S2-Entry 3.

Figure S 23. ATR-FT-IR spectrum of the polyamine produced from the hydrogenation of NBR. Table S2-Entry 3.

*Table S2-Entry 4*

**^1^H NMR** (CDCl_3_, 500 MHz): δ_H_ 5.39 (HC=CH), 4.96 (HC=CH), 2.58 (H_2_CNH_2_), 2.00 (H_2_CH=CH), 1.41 (CHCH_2_NH_2_ or CHCH=CH_2_), 1.29 (CHCH_2_), 0.87 (CH_3_).

**^13^C{^1^H} NMR** (CDCl_3_, 126 MHz): δ_c_ 132.0-128.1 (HC=CH), 44.6 (CH_2_-NH_2_), 40.6 (CHCH_2_NH_2_), 34.6, 32.8. 30.0 (CH_2_CH=CH), 31.3 (CHCH_2_),

**IR** (ATR-FTIR, cm^–1^): ν 3400 and 3286 (NH stretch), 2915m (C-H stretch), 1560 (N-H bend, C=C stretch), 1448S (C-C), 966sh (HC bend from internal [1,4-trans] olefin), 908s (HC bend from 1,2 vinyl olefin).

*
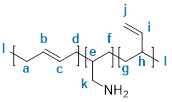
*

**b**

**c**

**i**

**e**

**h f**

**a**

**d**

**k**

**j**

Figure S 24. ^1^H NMR spectrum (CDCl_3_, 500 MHz, 298K) of the polyamine produced from the hydrogenation of NBR. Table S2-Entry 4.

*
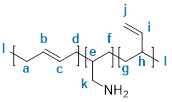
*

**k**

Figure S 25. ^13^C{^1^H} NMR spectrum (126 MHz, CDCl_3_, 298K) of the polyamine produced from the hydrogenation of NBR. Table S2-Entry 4.

*
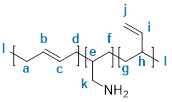
*

**k**

Figure S 26. ^1^H, ^13^C- HSQC NMR spectrum (CDCl_3_) of the polyamine produced from the hydrogenation of NBR. Table S2-Entry 4.

Figure S 27. ATR-FT-IR spectrum of the polyamine produced from the hydrogenation of NBR. Table S2-Entry 4.


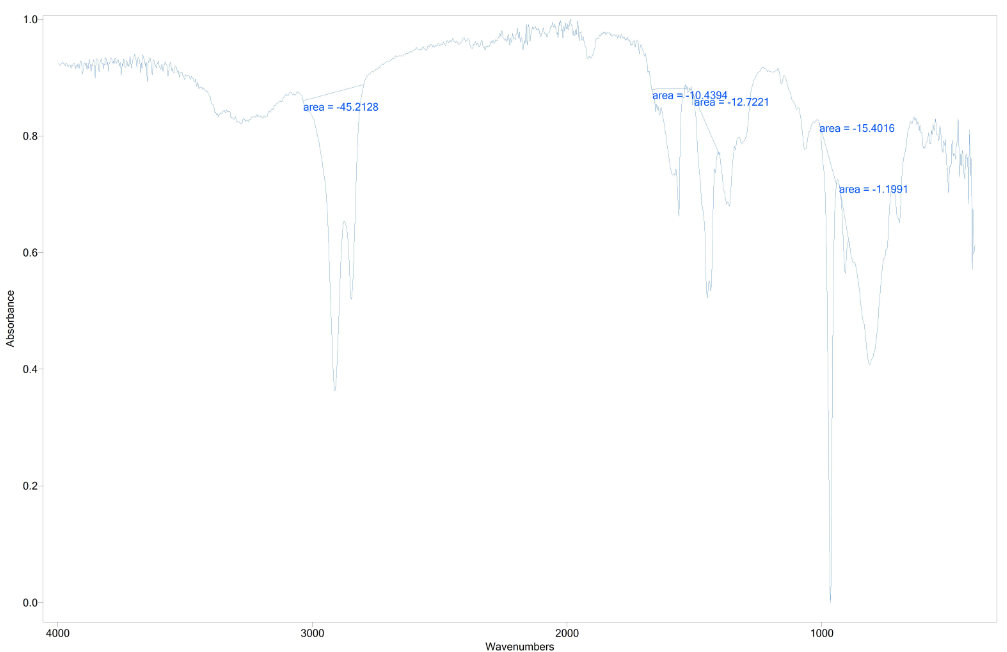


Figure S 28. ATR-FT-IR spectrum (signals integrated) of the polyamine produced from the hydrogenation of NBR. Table S2-Entry 4.

*Table S2-Entry 5.*

**IR** (ATR-FTIR, cm^–1^): ν 2922m (C-H stretch), 2237sh (C≡N), 1560S (C=C stretch), 1462m (C-C).

Figure S 29. ATR-FT-IR spectrum of the polyamine produced from the hydrogenation of NBR. Table S2-Entry 5. No presence of a double bond.

*Table S2-Entry 6*

**^1^H NMR** (CDCl_3_, 500 MHz): δ_H_ 5.39 (HC=CH), 4.96 (HC=CH), 2.58 (H_2_CNH_2_), 2.00 (H_2_CH=CH), 1.38 (CHCH_2_NH_2_ or CHCH=CH_2_), 1.29 (CHCH_2_).

**^13^C{^1^H} NMR** (CDCl_3_, 126 MHz): δ_c_ 132.0-128.1 (HC=CH), 44.7 (CH_2_-NH_2_), 40.6 (CHCH_2_NH_2_), 34.7, 32.8. 30.0 (CH_2_CH=CH), 31.3 (CHCH_2_).

**IR** (ATR-FTIR, cm^–1^): ν 3320 and 3298 (N-H stretch), 2908m (C-H stretch), 1564 (N-H bend, C=C stretch),1436S (C-C), 968sh (HC bend from internal [1,4-trans] olefin), 908s (HC bend from 1,2 vinyl olefin).

*
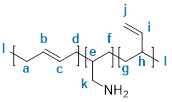
*

**b**

**c**

**i**

**j**

**e**

**h f**

**l**

**a**

**d**

**k**

Figure S 30. ^1^H NMR spectrum (CDCl_3_, 500 MHz, 298K) of the polyamine produced from the hydrogenation of NBR. Table S2-Entry 6.

*
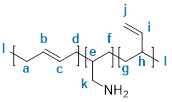
*

**k**

Figure S 31. ^13^C{^1^H} NMR spectrum (126 MHz, CDCl_3_, 298K) of the polyamine produced from the hydrogenation of NBR. Table S2-Entry 6.

Figure S 32. ATR-FT-IR spectrum of the polyamine produced from the hydrogenation of NBR. Table S2-Entry 6.

*Table S2-Entry 7*

**^1^H NMR** (CDCl_3_, 500 MHz): δ_H_ 5.42 (HC=CH), 4.99 (HC=CH), 2.61 (H_2_CNH_2_), 2.04 (H_2_CH=CH), 1.44 (CHCH_2_NH_2_ or CHCH=CH_2_), 1.33 (CHCH_2_), 0.88 (CH_3_)

**^13^C{^1^H} NMR** (CDCl_3_, 126 MHz): δ_c_ 132.1-128.1 (HC=CH), 44.7 (CH_2_-NH_2_), 40.5 (CHCH_2_NH_2_), 34.6, 32.8. 30.0 (CH_2_CH=CH), 31.3 (CHCH_2_),

**IR** (ATR-FTIR, cm^–1^): ν 3300-3352 (N-H stretch), 2912m (C-H stretch), 1562S (N-H bend, C=C stretch), 1433m(C-C), 1128 (C-N stretch), 954sh (HC bend from internal [1,4-trans] olefin), 908s (HC bend from 1,2 vinyl olefin).

*
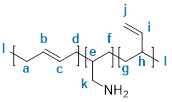
*

**b**

**c**

**i**

**e**

**h f**

**f**

**a**

**d**

**k**

**j**

Figure S 33. ^1^H NMR spectrum (CDCl_3_, 500 MHz, 298K) of the polyamine produced from the hydrogenation of NBR. Table S2-Entry 7.

*
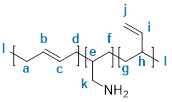
*

**k**

Figure S 34. ^13^C{^1^H} NMR spectrum (126 MHz, CDCl_3,_ 298K) of the polyamine produced from the hydrogenation of NBR. Table S2-Entry 7.

*
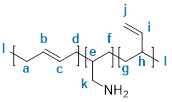
*

**k**

Figure S 35. ^1^H, ^13^C- HSQC NMR (CDCl_3_) spectrum of the polyamine produced from the hydrogenation of NBR. Table S2-Entry 7.

Figure S 36. ATR-FT-IR spectrum of the polyamine produced from the hydrogenation of NBR. Table S2-Entry 7.

*Table S2-Entry 8*

**^1^H NMR** (CDCl_3_, 500 MHz): δ_H_ 5.56-5.40 (HC=CH), 4.99 (HC=CH), 2.59 (H_2_CNH_2_ and HCCN), 2.04 (H_2_CH=CH), 1.44 (CHCH_2_NH_2_ or CHCH=CH_2_), 1.33 (CHCH_2_).

**^13^C{^1^H} NMR** (CDCl_3_, 126 MHz): δ_c_ 135.0-124.7 (HC=CH), 121.8 (CN), 44.7 (CH_2_-NH_2_), 40.5 (CHCH_2_NH_2_), 34.6, 32.7. 30.0 (CH_2_CH=CH), 31.3 (CHCH_2_).

**IR** (ATR-FTIR, cm^–1^): ν 3340 (N-H stretch), 2915m (C-H stretch), 2235 (C≡N), 1570S (N-H bend, C=C stretch), 1436m(C-C), 968sh (HC bend from internal [1,4-trans] olefin), 918s (HC bend from 1,2 vinyl olefin).

Figure S 37. ^1^H NMR spectrum (CDCl_3_, 500 MHz, 298K) of the polyamine produced from the hydrogenation of NBR. Table S2-Entry 8.

*
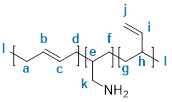
*
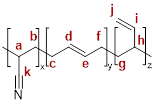


**k**

**k**

Figure S 38. ^13^C{^1^H} NMR spectrum (126 MHz, CDCl_3_, 298K) of the polyamine produced from the hydrogenation of NBR. Table S2-Entry 8.


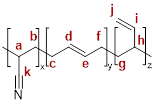
*
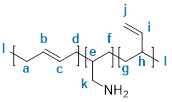
*

**k**

**k**

Figure S 39. ^1^H, ^13^C- HSQC NMR (CDCl_3_) spectrum of the polyamine produced from the hydrogenation of NBR. Table S2-Entry 8.

Figure S 40. ATR-FT-IR spectrum of the polyamine produced from the hydrogenation of NBR. Table S2-Entry 8.


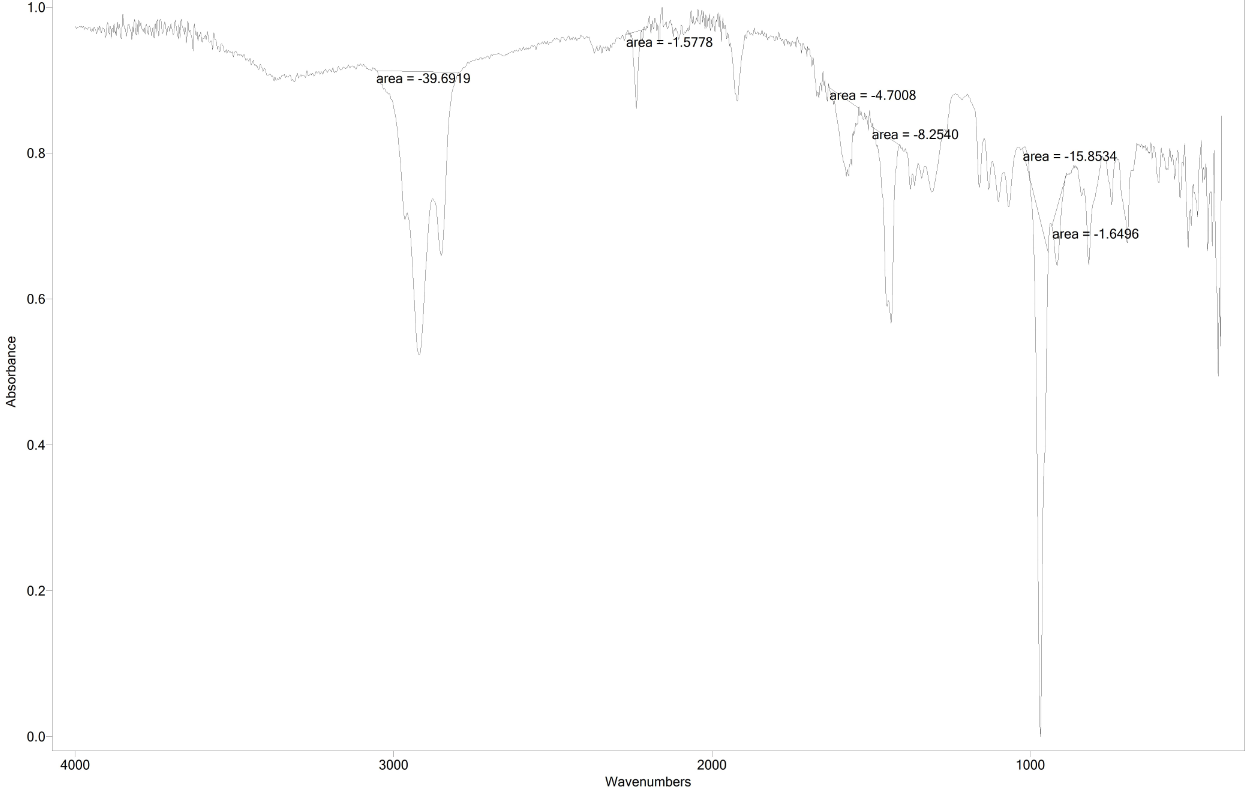


Figure S 41. ATR-FT-IR spectrum (integrated) of the polyamine produced from the hydrogenation of NBR. Table 1-Entry 8.

*Table S2-Entry 9*

**^1^H NMR** (CDCl_3_, 500 MHz): δ_H_ 5.30 (HC=CH), 4.97 (HC=CH), 2.59 (H_2_CNH_2_), 2.03 (H_2_CH=CH), 1.38 (CHCH_2_NH_2_ or CHCH=CH_2_), 1.34 (CHCH_2_).

**^13^C{^1^H} NMR** (CDCl_3_, 126 MHz): δ_c_ 132.1-128.1 (HC=CH), 44.7 (CH_2_-NH_2_), 40.6 (CHCH_2_NH_2_), 34.6, 32.8. 30.0 (CH_2_CH=CH), 31.3 (CHCH_2_).

**IR** (ATR-FTIR, cm^–1^): ν 3194 (N-H stretch), 2915m (C-H stretch), 1562S (N-H bend, C=C stretch), 1442m(C-C), 966sh (HC bend from internal [1,4-trans] olefin), 910s (HC bend from 1,2 vinyl olefin).

*
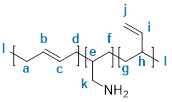
*

**b**

**c**

**i**

**e**

**h f**

**a**

**d**

**k**

**j**

Figure S 42. ^1^H NMR spectrum (CDCl_3_, 500 MHz, 298K) of the polyamine produced from the hydrogenation of NBR. Table S2-Entry 9.

*
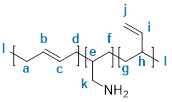
*

**k**

Figure S 43. ^13^C{^1^H} NMR spectrum (126 MHz, CDCl_3_, 298K) of the polyamine produced from the hydrogenation of NBR. Table S2-Entry 9.

*
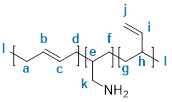
*

**k**

Figure S 44. ^1^H, ^13^C- HSQC NMR (CDCl_3_) spectrum of the polyamine produced from the hydrogenation of NBR. Table S2-Entry 9.

Figure S 45. ATR-FT-IR spectrum of the polyamine produced from the hydrogenation of NBR. Table S2-Entry 9.

*Table 2-Entry 10*

**^1^H NMR** (CDCl_3_, 500 MHz): δ_H_ 5.56-5.39 (HC=CH), 4.99 (HC=CH), 2.58 (H_2_CNH_2_ and HCCN), 2.04 (H_2_CH=CH), 1.40 (CHCH_2_NH_2_ or CHCH=CH_2_), 1.33 (CHCH_2_).

**^13^C{^1^H} NMR** (CDCl_3_, 126 MHz): δ_c_ 135.0-124.7 (HC=CH), 121.9 (CN), 44.6 (CH_2_-NH_2_), 40.5 (CHCH_2_NH_2_), 34.6, 32.6, 30.1 (CH_2_CH=CH), 31.3 (CHCH_2_).

**IR** (ATR-FTIR, cm^–1^): ν 2915m (C-H stretch), 2237 (C≡N), 1577S (N-H bend, C=C stretch), 1436m(C-C), 966sh (HC bend from internal [1,4-trans] olefin), 916s (HC bend from 1,2 vinyl olefin).

Figure S 46. ^1^H NMR spectrum (CDCl_3_, 500 MHz, 298K) of the polyamine produced from the hydrogenation of NBR. Table S2-Entry 10.


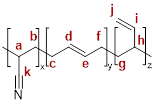
*
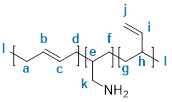
*

**k**

**k**

Figure S 47. ^13^C{^1^H} NMR spectrum (126 MHz, CDCl_3_, 298K) of the polyamine produced from the hydrogenation of NBR. Table S2-Entry 10.


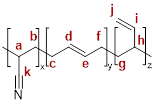
*
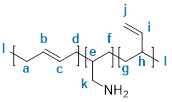
*

**k**

**k**

Figure S 48. ^1^H, ^13^C- HSQC NMR (CDCl_3_) spectrum of the polyamine produced from the hydrogenation of NBR. Table S2-Entry 10.

Figure S 49. ATR-FT-IR spectrum of the polyamine produced from the hydrogenation of NBR. Table S2-Entry 10.


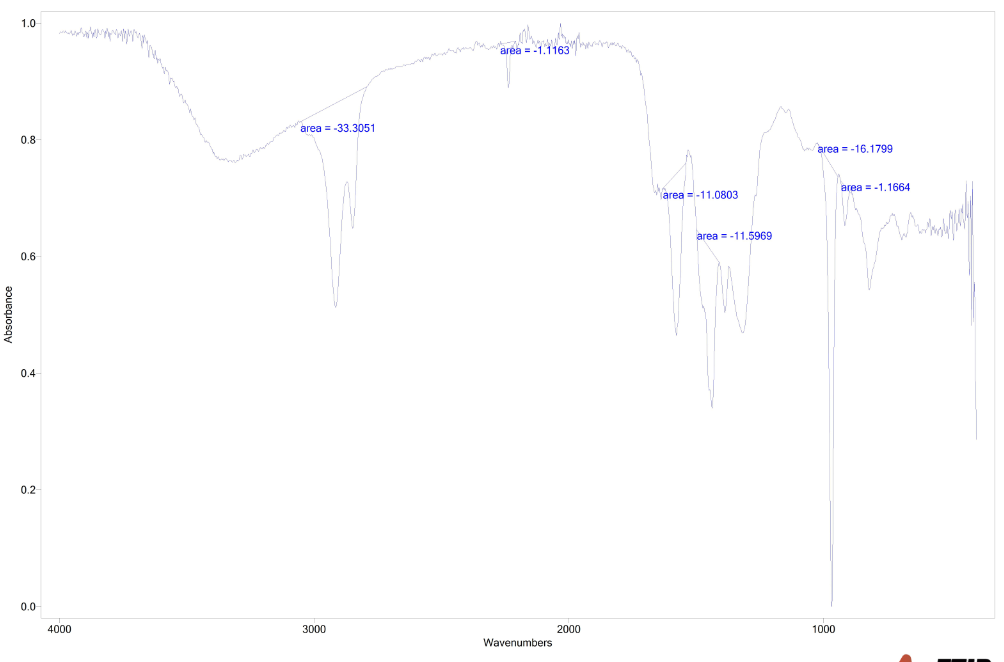


Figure S 50. ATR-FT-IR spectrum (integrated) of the polyamine produced from the hydrogenation of NBR. Table S2-Entry 10.

*Table S2-Entry 11*

**^1^H NMR** (CDCl_3_, 500 MHz): δ_H_ 5.52-5.42 (HC=CH), 5.00 (HC=CH), 2.60 (H_2_CNH_2_ and HCCN), 2.06 (H_2_CH=CH), 1.40 (CHCH_2_NH_2_ or CHCH=CH_2_), 1.33 (CHCH_2_).

**^13^C{^1^H} NMR** (CDCl_3_, 126 MHz): δ_c_ 132.7-124.7 (HC=CH), 121.9 (CN), 44.6 (CH_2_-NH_2_), 40.5 (CHCH_2_NH_2_), 35.1, 32.6. 30.1 (CH_2_CH=CH), 31.3 (CHCH_2_).

**IR** (ATR-FTIR, cm^–1^): ν 3246 (N-H stretch), 2914m (C-H stretch), 2237 (C≡N), 1562S (N-H bend, C=C stretch), 1448m(C-C), 966sh (HC bend from internal [1,4-trans] olefin), 914s (HC bend from 1,2 vinyl olefin).

Figure S 51. ^1^H NMR spectrum (CDCl_3_, 500 MHz, 298K) of the polyamine produced from the hydrogenation of NBR. Table S2-Entry 11.

*
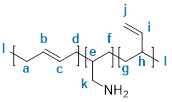
*
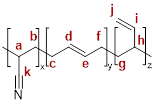


**k**

**k**

Figure S 52. ^13^C{^1^H} NMR spectrum (126 MHz, CDCl_3_, 298K) of the polyamine produced from the hydrogenation of NBR. Table S2-Entry 11.

*
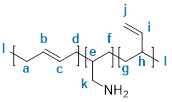
*
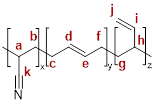


**k**

**k**

Figure S 53. ^1^H, ^13^C- HSQC NMR (DMSO-d6) spectrum of the polyamine produced from the hydrogenation of NBR. Table S2-Entry 11.

Figure S 54. ATR-FT-IR spectrum of the polyamine produced from the hydrogenation of NBR. Table S2-Entry 11.


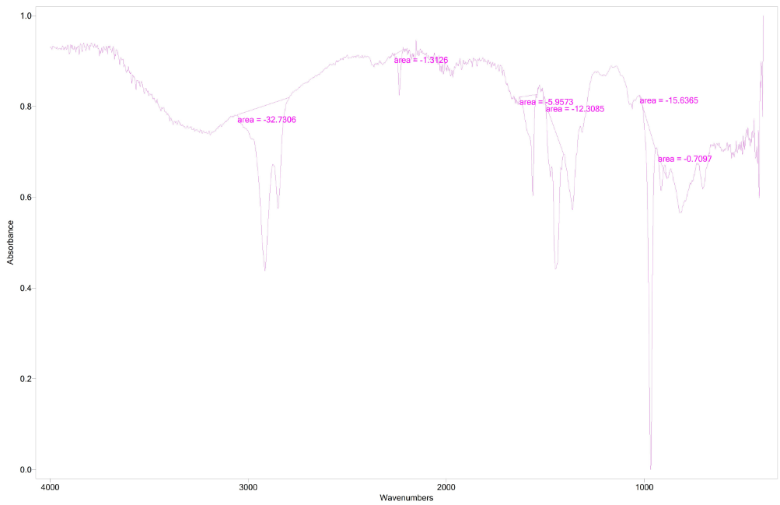


Figure S 55. ATR-FT-IR spectrum of the polyamine produced from the hydrogenation of NBR. Table S2-Entry 11.

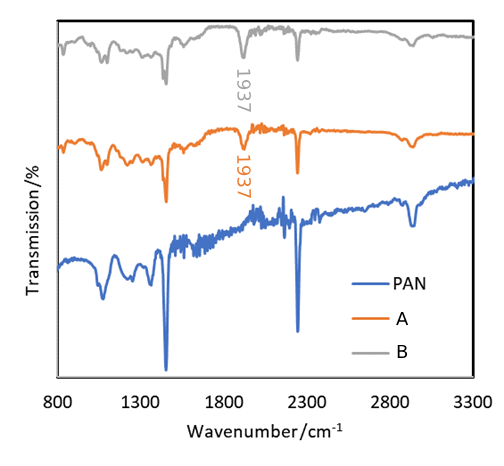


Figure S 56. ATR-FT-IR spectrum of polyacrylonitrile hydrogenation tests. Appearance of signals around 1900 cm^-1^ is suggestive of crosslinking.

**b**

**c**

**a**

**b**

**c**

**a**

Figure S 57. ^1^H NMR spectrum (CDCl_3_, 500 MHz, 298K) of the product isolated from the reaction of allyl cyanide and **Ru-1** after column purification with DCM:Hexane. Yield of 43% obtained by weight after purification.

# **S6 Full NMR Characterization of the polyol**


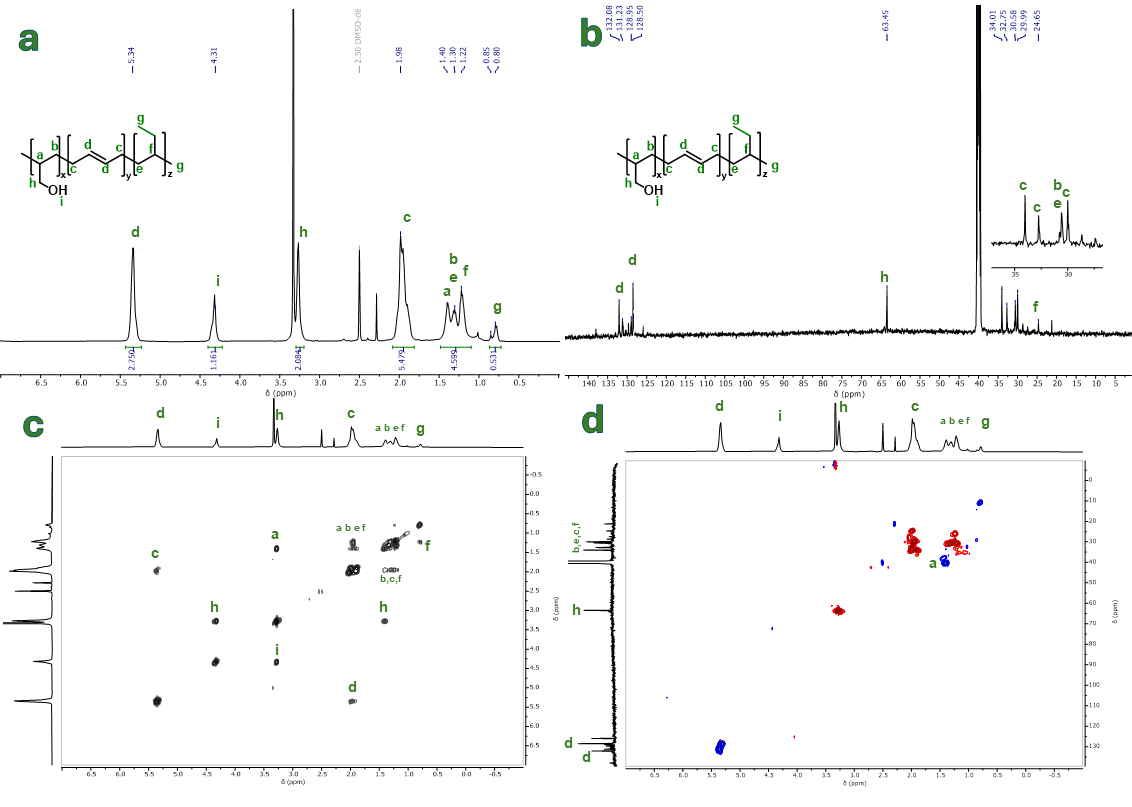


Figure S 58. Full NMR characterization of the Polyol obtained from NBR hydrogenation (Table 3, Entry 1) (a) ^1^H NMR in DMSO-d6. (b) ^13^C NMR in DMSO -d6. (c) ^1^H-^1^H COSY NMR in DMSO-d6. (d) ^1^H, ^13^C- HSQC NMR in DMSO-d6.

Table S 4. Optimisation of precatalyst choice for the hydrogenation of NBR to polyol.^a^

*Table S4. Entry 1*

**^1^H NMR** (CDCl_3_, 500 MHz): δ_H_ 5.54-5.37 (HC=CH), 5.06-4.94 (H_2_C=CH), 4.32 (OH), 3.28 (H_2_COH), 2.76 (HCCN), 2.24(H_2_CH=CH), 2.01 (H_2_CH=CH), 1.57 (CH_2_CHCN) 1.42 (CHCH_2_OH), 1.33 (CH=CHCH_2_CH_2_).

**^13^C{^1^H} NMR** (CDCl_3_, 126 MHz): δ_c_ 132.7-124.7 (HC=CH), 122.4 (CN), 63.4 (CH_2_-OH), 34.7, 32.4. 30.8 (CH_2_CH=CH), 31.2 (CHCH_2_).

**IR** (ATR-FTIR, cm^–1^): ν 3400 (O-H stretch), 2918m (C-H stretch), 2241 (CN), 1670 (C=C stretch), 1433m (C-C), 1029 (C-O stretch), 968sh (HC bend from internal [1,4-trans] olefin), 923s (HC bend from 1,2 vinyl olefin).


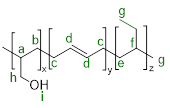


**h**

**h**

**a**

**i**

Figure S 59. ^1^H NMR spectrum (DMSO-d6, 500 MHz, 298K) of the polyol produced from the hydrogenation of NBR. Table S4– Entry 1.


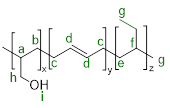

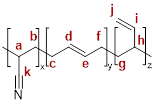


**h**

**k**

Figure S 60. ^13^C{^1^H} NMR spectrum (126 MHz, DMSO-d6, 298K) of the polyol produced from the hydrogenation of NBR. Table S4– Entry 1.

Figure S 61. ATR-FT-IR spectrum of the polyol produced from the hydrogenation of NBR. Table S4– Entry 1.

*Table S4. Entry 2*

**^1^H NMR** (CDCl_3_, 500 MHz): δ_H_ 5.54-5.35 (HC=CH), 4.37 (OH), 3.27 (H_2_COH), 2.73 (HCCN), 2.21(H_2_CH=CH), 1.98 (H_2_CH=CH), 1.55 (CH_2_CHCN) 1.41 (CHCH_2_OH), 1.31 (CH=CHCH_2_CH_2_).

**^13^C{^1^H} NMR** (CDCl_3_, 126 MHz): δ_c_ 134.9-129.9 (HC=CH), 122.47 (C≡N), 63.4 (CH_2_-OH), 34.7, 34.0 (CH_2_CH=CH), 31.2 (CHCH_2_).

**IR** (ATR-FTIR, cm^–1^): ν 2918m (C-H stretch), 2241 (C≡N), 1670 (C=C stretch), 1433m (C-C), 1029 (C-O stretch), 968sh (HC bend from internal [1,4-trans] olefin, 923s (HC bend from 1,2 vinyl olefin).


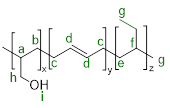


**h**

**i**

**a**

**h**

Figure S 62. ^1^H NMR spectrum (DMSO-d6, 500 MHz, 298K) of the polyol produced from the hydrogenation of NBR. Table S4– Entry 2.


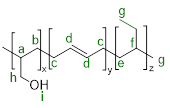

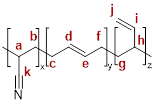


**k**

**h**

Figure S 63. ^13^C{^1^H} NMR spectrum (126 MHz, DMSO-d6, 298K) of the polyol produced from the hydrogenation of NBR. Table S4– Entry 2.

*Table S4. Entry 3*

**^1^H NMR** (CDCl_3_, 500 MHz): δ_H_ 5.34 (HC=CH), 4.35 (OH), 3.26 (H_2_COH), 1.98 (H_2_CH=CH), 1.41 (CHCH_2_OH), 1.31 (CH=CHCH_2_CH_2_), 1.22 (CH_2_CH_3_), 0.80 (CH_2_CH_3_).

**^13^C{^1^H} NMR** (CDCl_3_, 126 MHz): δ_c_ 132.1-128.5 (HC=CH), 63.4 (CH_2_-OH), 34.0, 34.0, 30.0 (CH_2_CH=CH), 31.2 (CH_2_).

**IR** (ATR-FTIR, cm^–1^): ν 3400 (O-H stretch), 2916m (C-H stretch), 1433m(C-C), 1033 (C-O stretch), 966sh (HC bend from internal [1,4-trans] olefin).


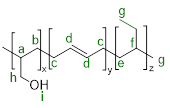

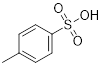


**h**

**i**

Figure S 64. ^1^H NMR spectrum (DMSO-d6, 500 MHz, 298K) of the polyol produced from the hydrogenation of NBR. Table S4– Entry 3.


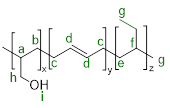


**h**

Figure S 65. ^13^C{^1^H} NMR spectrum (126 MHz, DMSO-d6, 298K) of the polyol produced from the hydrogenation of NBR. Table S4– Entry 3.

Figure S 66. ATR-FT-IR spectrum of the polyol produced from the hydrogenation of NBR. Table S4– Entry 3.

Figure S 67. (a) TGA thermogram of the polyol produced from the hydrogenation of NBR. Table S4– Entry 3. Onset temperature of 409 °C.

**1**

**3**

**2**

Figure S 68. GPC data of the polyol produced from the hydrogenation of NBR. Table S4– Entry 3. (1) M_n_: 577031 g/mol, PDI: 1.76 (2) M_n_: 5476 g/mol, PDI: 2.19, (3) M_n_: 425 g/mol, PDI: 1.25. GPC of the isolated material showed a bimodal chromatogram of M_n_= ~577 k and ~5k, confirming that the isolated material is indeed a polymer. Running a GPC of the starting NBR polymer (Figure S6) also showed a bimodal chromatogram of M_n_= ~2.1 M and ~27 k. We speculate that the difference in molecular weights arises due to the difference in hydrodynamic volume of polyols and NBR.


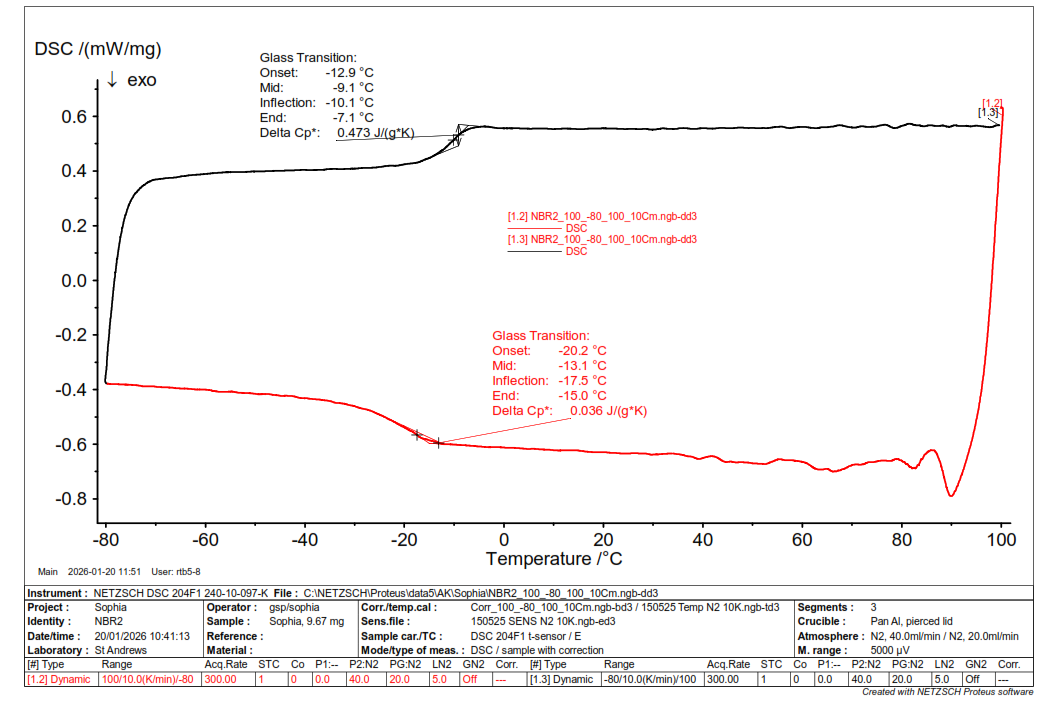


Figure S 69. DSC of polyol obtained from NBR hydrogenation. Amorphous material with a glass transition of -12.9 °C.

Table S 5 Elemental analysis of polyol obtained from NBR hydrogenation.

C,71.50; H,9.69; N, 0.98; O, 13.99

*Table S4. Entry 4*

**IR** (ATR-FTIR, cm^–1^): ν 3400 (O-H stretch), 2916m (C-H stretch), 1575 and 1539 (C=C stretch), 1458m(C-C), 1039 (C-O stretch), 966sh (HC bend from internal [1,4-trans] olefin).

Figure S 70. ATR-FT-IR spectrum of the polyol produced from the hydrogenation of NBR. Table S4– Entry 4.

*Table S4. Entry 5*

**IR** (ATR-FTIR, cm^–1^): ν 3400 (O-H stretch), 2920m (C-H stretch), 1458m(C-C), 1037 (C-O stretch), 966sh (HC bend from internal [1,4-trans] olefin).

Figure S 71. ATR-FT-IR spectrum of the polyol produced from the hydrogenation of NBR. Table S4– Entry 5.

*Table S4. Entry 6*

**^1^H NMR** (CDCl_3_, 500 MHz): δ_H_ 5.54 (HC=CH), 5.08 (H_2_C=CH), 2.78 (CHC≡N), 2.29 (H_2_CH=CH), 2.04 (CHCHC=CH_2_)_,_ 1.59 (CH_2_CHC≡N),1.24.

**^13^C{^1^H} NMR** (CDCl_3_, 126 MHz): δ_c_ 133.9-125.9 (HC=CH), 122.4 (CN), 34.68, 32.3 (CH_2_CH=CH), 31.2 (CHCH_2_).

**IR** (ATR-FTIR, cm^–1^): ν 2929m (C-H stretch), 2237sh (C≡N), 1652S (C=C stretch), 1436m(C-C), 968sh (HC bend from internal [1,4-trans] olefin), 916s (HC bend from 1,2 vinyl olefin).


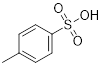


**h**

**a**

THF

THF

Figure S 72. ^1^H NMR spectrum (DMSO-d6, 500 MHz, 298K) of the polyol produced from the hydrogenation of NBR. Table S4– Entry 6.


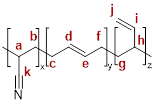


**k**

THF

THF

Figure S 73. ^13^C{^1^H} NMR spectrum (126 MHz, DMSO-d6, 298K) of the polyol produced from the hydrogenation of NBR. Table S4– Entry 6.

Figure S 74. ATR-FT-IR spectrum of the polyol produced from the hydrogenation of NBR. Table S4– Entry 6.

*Table S4. Entry 7*

**^1^H NMR** (CDCl_3_, 500 MHz): δ_H_ 5.55 (HC=CH), 5.08 (H_2_C=CH), 2.80 (CHC≡N), 2.29 (H_2_CH=CH), 2.05 (CHCHC=CH_2_)_,_ 1.58 (CH_2_CHC≡N),1.24.

**^13^C{^1^H} NMR** (CDCl_3_, 126 MHz): δ_c_ 132.7-125.9 (HC=CH), 122.4 (CN), 34.6, 29.88 (CH_2_CH=CH), 30.7 (CHCH_2_).

**IR** (ATR-FTIR, cm^–1^): ν 2927m (C-H stretch), 2237sh (C≡N), 1635S (N-H bend, C=C stretch), 1448m(C-C), 968sh (C=C).


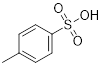


**h**

**a**

THF

THF

Figure S 75. ^1^H NMR spectrum (DMSO-d6, 500 MHz, 298K) of the polyol produced from the hydrogenation of NBR. Table S4– Entry 7.


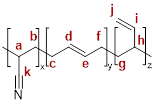


THF

THF

**k**

Figure S 76. ^13^C{^1^H} NMR spectrum (126 MHz, DMSO-d6, 298K) of the polyol produced from the hydrogenation of NBR. Table S4– Entry 7.

Figure S 77. ATR-FT-IR spectrum of the polyol produced from the hydrogenation of NBR. Table S4– Entry 7.

*Table S4. Entry 8*

**^1^H NMR** (CDCl_3_, 500 MHz): δ_H_ 5.55 (HC=CH), 5.05 (H_2_C=CH), 2.80 (CHC≡N), 2.28 (H_2_CH=CH), 2.05 (CHCHC=CH_2_)_,_ 1.58 (CH_2_CHC≡N),1.24, 0.84.

**^13^C{^1^H} NMR** (CDCl_3_, 126 MHz): δ_c_ 133.9-125.9 (HC=CH), 122.4 (CN), 34.60, 32.3, 29.7 (CH_2_CH=CH), 30.7 (CHCH_2_).

**IR** (ATR-FTIR, cm^–1^): ν 2922m (C-H stretch), 2235sh (C≡N), 1637S (C=C stretch), 1442m(C-C), 972sh (HC bend from internal [1,4-trans] olefin), 908sh (HC bend from 1,2 vinyl olefin).


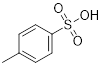


**h**

THF

**a**

THF

Figure S 78. ^1^H NMR spectrum (DMSO-d6, 500 MHz, 298K) of the polyol produced from the hydrogenation of NBR. Table S4– Entry 8.


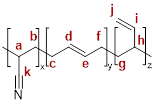


THF

THF

**k**

Figure S 79.^13^C{^1^H} NMR spectrum (126 MHz, DMSO-d6) of the polyol produced from the hydrogenation of NBR. Table S4– Entry 8.

Figure S 80. ATR-FT-IR spectrum of the polyol produced from the hydrogenation of NBR. Table S4– Entry 8.

*Table S4. Entry 9*

**IR** (ATR-FTIR, cm^–1^): ν 2926m (C-H stretch), 1446m(C-C), 877sh (C=C).

Figure S 81. ATR-FT-IR spectrum of the polyol produced from the hydrogenation of NBR. Table S4– Entry 9. No absorption band is observed at ~2200 cm⁻¹, corresponding to the nitrile stretching vibration, indicating that the nitrile functionality has reacted. Additionally, the characteristic signals associated with the double bond (972 and 908 cm⁻¹) are also absent. Together with the lack of a nitrile crosslinking-related signal at ~1900 cm⁻¹, these observations suggest that the insoluble material was formed through crosslinking via the double bonds.

Table S 6. Optimisation of reaction conditions for the hydrogenation of NBR to polyols using **Ru-3**.^a^

^a^Reaction conditions: NBR (0.94 mmol or 100 mg), THF (1.5 mL), H_2_O (0.5 mL), 150 °C, 40 bar.

^b^Conversion is estimated by the consumption of C**H**CN signal, estimated by ^1^H NMR (2.8 ppm) spectra taken in DMSO-d6.

^c^Yields are based on the weight of isolated product.

^d^Yield is determined by ^1^H NMR spectroscopy in DMSO-d6 by the integration of C**H_2_**OH signals.

^e^100 °C. ^f^ 20 bar. ^g^ 1 gram NBR.

*Table S6 Entry 1*

**^1^H NMR** (CDCl_3_, 500 MHz): δ_H_ 5.33 (HC=CH), 4.34 (OH), 3.27 (H_2_COH), 1.98 (H_2_CH=CH), 1.39 (CHCH_2_OH), 1.32 (CH=CHCH_2_CH_2_), 1.22 (CH_2_CH_3_). 0.77 (CH_2_CH_3_).

**^13^C{^1^H} NMR** (CDCl_3_, 126 MHz): δ_c_ 132.1-125.9 (HC=CH), 63.5 (CH_2_-OH), 34.0, 32.7, 29.99 (CH_2_CH=CH), 30.5 (CH_2_).

**IR** (ATR-FTIR, cm^–1^): ν 3373 (O-H stretch), 2916m (C-H stretch), 1454m(C-C), 1649S (C=C stretch), 1033 (C-O stretch), 966sh (HC bend from internal [1,4-trans] olefin).


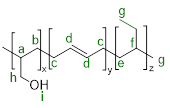

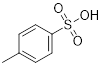


**h**

**i**

Figure S 82. ^1^H NMR spectrum (DMSO-d6, 500 MHz, 298K) of the polyol produced from the hydrogenation of NBR. Table S3– Entry 1.


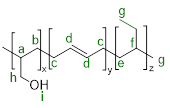


THF

**h**

THF

Figure S 83. ^13^C{^1^H} NMR spectrum (126 MHz, DMSO-d6, 298K) of the polyol produced from the hydrogenation of NBR. Table S6– Entry 1.

Figure S 84. ATR-FT-IR spectrum of the polyol produced from the hydrogenation of NBR. Table S6– Entry 1.

*Table S3 Entry 2*

**^1^H NMR** (CDCl_3_, 500 MHz): δ_H_ 5.53 (HC=CH), 5.04 (H_2_C=CH), 2.80 (CHC≡N), 2.29 (H_2_CH=CH), 2.04 (CHCHC=CH_2_)_,_ 1.59 (CH_2_CHC≡N),1.24.

**^13^C{^1^H} NMR** (CDCl_3_, 126 MHz): δ_c_ 132.7-125.9 (HC=CH), 122.4 (CN), 34.64, 32.3, 29.88 (CH_2_CH=CH), 30.7 (CHCH_2_).

**IR** (ATR-FTIR, cm^–1^): ν 2926m (C-H stretch), 2235sh (C≡N), 1645S (C=C stretch), 1444m(C-C), 968sh (HC bend from internal [1,4-trans] olefin), 914s (HC bend from 1,2 vinyl olefin).


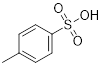


**h**

**a**

THF

THF

Figure S 85. ^1^H NMR spectrum (DMSO-d6, 500 MHz, 298K) of the polyol produced from the hydrogenation of NBR. Table S6– Entry 2.


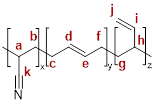


THF

THF

**k**

Figure S 86. ^13^C{^1^H} NMR spectrum (126 MHz, DMSO-d6, 298K) of the polyol produced from the hydrogenation of NBR. Table S3– Entry 2.

Figure S 87. ATR-FT-IR spectrum of the polyol produced from the hydrogenation of NBR. Table S6– Entry 2.

*Table S6 Entry 3*

**^1^H NMR** (CDCl_3_, 500 MHz): δ_H_ 5.34 (HC=CH), 4.31 (OH), 3.27 (H_2_COH), 1.98 (H_2_CH=CH), 1.39 (CHCH_2_OH), 1.31 (CH=CHCH_2_CH_2_), 1.23 (CH_2_CH_3_). 0.79 (CH_2_CH_3_).

**^13^C{^1^H} NMR** (CDCl_3_, 126 MHz): δ_c_ 131.6-127.9 (HC=CH), 63.1 (CH_2_-OH), 33.6, 32.3, 29.9 (CH_2_CH=CH), 30.7(CH_2_).

**IR** (ATR-FTIR, cm^–1^): ν 3470 (O-H stretch), 2920m (C-H stretch), 1442m(C-C), 1029 (C-O stretch), 966sh (HC bend from internal [1,4-trans] olefin).


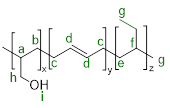

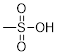


**h**

**i**

Figure S 88. ^1^H NMR spectrum (DMSO-d6, 500 MHz, 298K) of the polyol produced from the hydrogenation of NBR. Table S6– Entry 3.


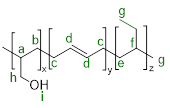


**h**

Figure S 89. ^13^C{^1^H} NMR spectrum (126 MHz, DMSO-d6, 298K) of the polyol produced from the hydrogenation of NBR. Table S6– Entry 3.

Figure S 90. ATR-FT-IR spectrum of the polyol produced from the hydrogenation of NBR. Table S6– Entry 3.

*Table S6 Entry 4*

**^1^H NMR** (CDCl_3_, 500 MHz): δ_H_ 5.34 (HC=CH), 4.31 (OH), 3.27 (H_2_COH), 1.98 (H_2_CH=CH), 1.42 (CHCH_2_OH), 1.31 (CH=CHCH_2_CH_2_), 1.24 (CH_2_CH_3_). 0.78 (CH_2_CH_3_).

**^13^C{^1^H} NMR** (CDCl_3_, 126 MHz): δ_c_ 132.1-128.5 (HC=CH), 63.5 (CH_2_-OH), 34.0, 32.7, 29.7 (CH_2_CH=CH), 30.6 (CH_2_).

**IR** (ATR-FTIR, cm^–1^): ν 3342 (O-H stretch), 2916m (C-H stretch), 1436m(C-C), 1029 (C-O stretch), 966sh (HC bend from internal [1,4-trans] olefin).


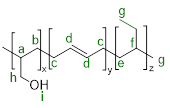

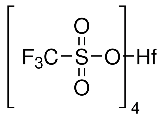


**i**

**h**

Figure S 91. ^1^H NMR spectrum (DMSO-d6, 500 MHz, 298K) of the polyol produced from the hydrogenation of NBR. Table S6– Entry 4.


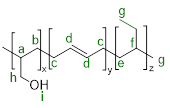


**h**

Figure S 92. ^13^C{^1^H} NMR spectrum (126 MHz, DMSO-d6, 298K) of the polyol produced from the hydrogenation of NBR. Table S6– Entry 4.

Figure S 93. ATR-FT-IR spectrum of the polyol produced from the hydrogenation of NBR. Table S6– Entry 4.

*Table S6 Entry 5*

**^1^H NMR** (CDCl_3_, 500 MHz): δ_H_ 5.55-5.40 (HC=CH), 5.05 (H_2_C=CH), 2.79 (CHC≡N), 2.29 (H_2_CH=CH), 2.04 (CHCHC=CH_2_)_,_ 1.59 (CH_2_CHC≡N),1.23.

**^13^C{^1^H} NMR** (CDCl_3_, 126 MHz): δ_c_ 138.1-126.0 (HC=CH), 122.4 (CN), 34.6, 32.3, 29.9 (CH_2_CH=CH), 30.9 (CHCH_2_).

**IR** (ATR-FTIR, cm^–1^): ν 2924m (C-H stretch), 2237sh (C≡N), 1642 (C=C stretch), 1440m(C-C), 966sh (HC bend from internal [1,4-trans) olefin).


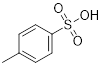


**h**

**a**

Figure S 94. ^1^H NMR spectrum (DMSO-d6, 500 MHz, 298K) of the polyol produced from the hydrogenation of NBR. Table S6– Entry 5.


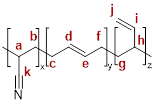


**k**

THF

THF

Figure S 95. ^13^C{^1^H} NMR spectrum (126 MHz, DMSO-d6, 298K) of the polyol produced from the hydrogenation of NBR. Table S6– Entry 5.

**1,4 trans**

**olefins**

**C-C**

**bending**

**C≡N**

**stretch**

**CH**

**stretch**

Figure S 96. ATR-FT-IR spectrum of the polyol produced from the hydrogenation of NBR. Table S6– Entry 5

*Table S6 Entry 6*

**^1^H NMR** (CDCl_3_, 500 MHz): δ_H_ 5.54-5.34 (HC=CH), 5.04-4.93 (H_2_C=CH), 4.32 (OH), 3.27 (H_2_COH), 2.73 (HCCN), 2.29 (H_2_CH=CH), 1.99 (H_2_CH=CH), 1.57 (CH_2_CHCN) 1.43 (CHCH_2_OH), 1.22 (CH=CHCH_2_CH_2_), 0.80 (CH_3_)

**^13^C{^1^H} NMR** (CDCl_3_, 126 MHz): δ_c_ 138.1-126.0 (HC=CH), 63.5 (CH_2_-OH), 34.7, 32.8. 30.0 (CH_2_CH=CH), 30.6 (CHCH_2_).


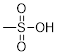

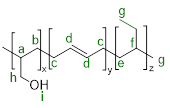
**IR** (ATR-FTIR, cm^–1^): ν 2918m (C-H stretch), 2241 (C≡N), 1435m(C-C), 1033 (C-O stretch), 968sh (HC bend from internal [1,4-trans] olefin) .

**a**

**h**

**i**

**h**

Figure S 97. ^1^H NMR spectrum (DMSO-d6, 500 MHz, 298K) of the polyol produced from the hydrogenation of NBR. Table S6– Entry 6.


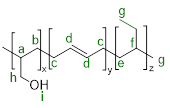

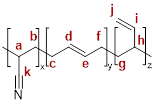


**h**

Figure S 98. ^13^C{^1^H} NMR spectrum (126 MHz, DMSO-d6) of the polyol produced from the hydrogenation of NBR. Table S6– Entry 6.

Figure S 99. ATR-FT-IR spectrum of the polyol produced from the hydrogenation of NBR. Table S6– Entry 6.

*Table S6 Entry 7*

**^1^H NMR** (CDCl_3_, 500 MHz): δ_H_ 5.54-5.39 (HC=CH), 3.31 (H_2_COH), 2.77 (HCCN), 2.26 (H_2_CH=CH), 2.04 (H_2_CH=CH), 1.57 (CH_2_CHCN) 1.41 (CHCH_2_OH), 1.23 (CH=CHCH_2_CH_2_), 0.80 (CH_3_)

**^13^C{^1^H} NMR** (CDCl_3_, 126 MHz): δ_c_ 138.1-126.0 (HC=CH), 122.4 (CN), 34.63, 32.47, 29.65 (CH_2_CH=CH), 30.7 (CHCH_2_).

**IR** (ATR-FTIR, cm^–1^): ν 2943m (C-H stretch), 2241 (C≡N), 1436m(C-C), 1024 (C-O stretch).


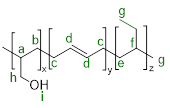

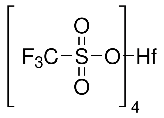


**a**

**h**

Figure S 100

^1^H NMR spectrum (DMSO-d6, 500 MHz, 298K) of the polyol produced from the hydrogenation of NBR. Table S6– Entry 7.


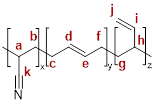


**k**

Figure S 101. ^13^C{^1^H} NMR spectrum (126 MHz, DMSO-d6, 298K) of the polyol produced from the hydrogenation of NBR. Table S6– Entry 7. Poor solubility.

Figure S 102. ATR-FT-IR spectrum of the polyol produced from the hydrogenation of NBR. Table S6– Entry 7.

*Table S6 Entry 8*

**^1^H NMR** (CDCl_3_, 500 MHz): δ_H_ 5.35 (HC=CH), 4.94 (H_2_C=CH), 4.32 (OH), 3.26 (H_2_COH), 1.99 (H_2_CH=CH), 1.39 (CHCH_2_OH), 1.31 (CH=CHCH_2_CH_2_), 1.21 (CH_2_CH_3_). 0.80 (CH_2_CH_3_).

**^13^C{^1^H} NMR** (CDCl_3_, 126 MHz): δ_c_ 132.1-126.0 (HC=CH), 63.4 (CH_2_-OH), 34.0, 32.7, 30.0 (CH_2_CH=CH), 30.6 (CH_2_).

**IR** (ATR-FTIR, cm^–1^): ν 3373 (O-H stretch), 2912m (C-H stretch), 1436m(C-C), 1654S (C=C stretch), 1035 (C-O stretch), 966sh (HC bend from internal [1,4-trans] olefin).


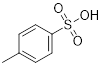


**g**

**h**

**i**

Figure S 103. ^1^H NMR spectrum (DMSO-d6, 500 MHz, 298K) of the polyol produced from the hydrogenation of NBR. Table S6– Entry 8. 84% of initial 1,2 vinyl olefins remain unreacted.

**h**

THF

THF

Figure S 104. ^13^C{^1^H} NMR spectrum (126 MHz, DMSO-d6, 298K) of the polyol produced from the hydrogenation of NBR. Table S6– Entry 8.

Figure S 105. ATR-FT-IR spectrum of the polyol produced from the hydrogenation of NBR. Table S6– Entry 8.

*Table S6 Entry 9*

**^1^H NMR** (CDCl_3_, 500 MHz): δ_H_ 5.34 (HC=CH), 4.93 (H_2_C=CH), 4.31 (OH), 3.26 (H_2_COH), 1.99 (H_2_CH=CH), 1.39 (CHCH_2_OH), 1.30 (CH=CHCH_2_CH_2_), 1.24 (CH_2_CH_3_). 0.79 (CH_2_CH_3_).

**^13^C{^1^H} NMR** (CDCl_3_, 126 MHz): δ_c_ 132.1-128.5 (HC=CH), 63.5 (CH_2_-OH), 34.0, 32.7, 30.0 (CH_2_CH=CH), 30.6 (CH_2_).

**IR** (ATR-FTIR, cm^–1^): ν 3375 (O-H stretch), 2914m (C-H stretch), 1641S (C=C stretch), 1450m(C-C), 1045 (C-O stretch), 966sh (HC bend from internal [1,4-trans] olefin).


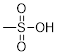


**g**

**h**

**i**

Figure S 106. ^1^H NMR spectrum (DMSO-d6, 500 MHz, 298K) of the polyol produced from the hydrogenation of NBR. Table S6– Entry 9. 100% of initial 1,2 vinyl olefins remain unreacted.

**h**

Figure S 107. ^13^C{^1^H} NMR spectrum (126 MHz, DMSO-d6, 298K) of the polyol produced from the hydrogenation of NBR. Table S6– Entry 9.

Figure S 108. ATR-FT-IR spectrum of the polyol produced from the hydrogenation of NBR. Table S6– Entry 9.

*Table S6 Entry 10*

**^1^H NMR** (CDCl_3_, 500 MHz): δ_H_ 5.54-5.40 (HC=CH), 2.78 (CHC≡N), 2.29 (H_2_CH=CH), 2.04 (CHCHC=CH_2_)_,_ 1.57 (CH_2_CHC≡N), (CH_2_CH_3_)1.23, 0.82 (CH_2_CH_3_).

**^13^C{^1^H} NMR** (CDCl_3_, 126 MHz): δ_c_ 132.7-126.0 (HC=CH), 122.4 (CN), 34.6, 32.3, 29.6 (CH_2_CH=CH), 30.7 (CHCH_2_).

**IR** (ATR-FTIR, cm^–1^): ν 2929m (C-H stretch), 2235sh (C≡N), 1714S (C=C stretch), 1436m(C-C), 968sh (HC bend from internal [1,4-trans] olefin), 916s (HC bend from 1,2 vinyl olefin).


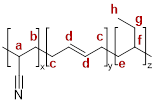

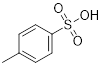


**a**

Figure S 109. ^1^H NMR spectrum (DMSO-d6, 500 MHz, 298K) of the polyol produced from the hydrogenation of NBR. Table S3– Entry 10.


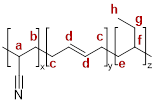


**k**

**k**

Figure S 110. ^13^C{^1^H} NMR spectrum (126 MHz, DMSO-d6, 298K) of the polyol produced from the hydrogenation of NBR. Table S6– Entry 10.

Figure S 111. ATR-FT-IR spectrum of the polyol produced from the hydrogenation of NBR. Table S6– Entry 10.

*Table S6 Entry 11*

**^1^H NMR** (CDCl_3_, 500 MHz): δ_H_ 5.34 (HC=CH), 4.32 (OH), 3.27 (H_2_COH), 1.98 (H_2_CH=CH), 1.39 (CHCH_2_OH), 1.30 (CH=CHCH_2_CH_2_), 1.24 (CH_2_CH_3_). 0.79 (CH_2_CH_3_).

**^13^C{^1^H} NMR** (CDCl_3_, 126 MHz): δ_c_ 132.6-128.5 (HC=CH), 63.4 (CH_2_-OH), 34.0, 32.7, 30.6 (CH_2_CH=CH), 31.17 (CH_2_).


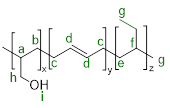
**IR** (ATR-FTIR, cm^–1^): ν 3365 (O-H stretch), 2918m (C-H stretch), 1577S (C=C stretch), 1433m(C-C), 1037 (C-O stretch), 964sh (HC bend from internal [1,4-trans] olefin).
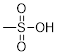


**h**

**i**

Figure S 112. ^1^H NMR spectrum (DMSO-d6, 500 MHz, 298K) of the polyol produced from the hydrogenation of NBR. Table S6– Entry 11.


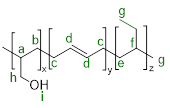


THF

THF

**h**

Figure S 113. ^13^C{^1^H} NMR spectrum (126 MHz, DMSO-d6, 298K) of the polyol produced from the hydrogenation of NBR. Table S6– Entry 11.

Figure S 114. ATR-FT-IR spectrum of the polyol produced from the hydrogenation of NBR. Table S6– Entry 11.

*Table S6 Entry 12*

**^1^H NMR** (CDCl_3_, 500 MHz): δ_H_ 5.34 (HC=CH), 4.33 (OH), 3.26 (H_2_COH), 1.98 (H_2_CH=CH), 1.39 (CHCH_2_OH), 1.33 (CH=CHCH_2_CH_2_), 1.23 (CH_2_CH_3_). 0.79 (CH_2_CH_3_).

**^13^C{^1^H} NMR** (CDCl_3_, 126 MHz): δ_c_ 132.1-128.4 (HC=CH), 63.9 (CH_2_-OH), 34.0, 32.7, 30.0 (CH_2_CH=CH), 30.6 (CH_2_).

**IR** (ATR-FTIR, cm^–1^): ν 3385 (O-H stretch), 2916m (C-H stretch), 1652S (C=C stretch), 1433m(C-C), 1045 (C-O stretch), 968sh (HC bend from internal [1,4-trans] olefin).


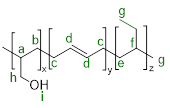

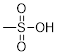


**h**

**i**

Figure S 115. ^1^H NMR spectrum (DMSO-d6, 500 MHz, 298K) of the polyol produced from the hydrogenation of NBR. Table S6– Entry 12.


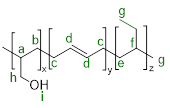


**h**

Figure S 116. ^13^C{^1^H} NMR spectrum (126 MHz, DMSO-d6, 298K) of the polyol produced from the hydrogenation of NBR. Table S6– Entry 12.

Figure S 117. ATR-FT-IR spectrum of material obtained from hydrogenation of Nitrile Butadiene Rubber (OH-NBR) corresponding to Table S6– Entry 12.

*Table S6 Entry 13*

**^1^H NMR** (CDCl_3_, 500 MHz): δ_H_ 5.34 (HC=CH), 4.32 (OH), 3.27 (H_2_COH), 1.98 (H_2_CH=CH), 1.39 (CHCH_2_OH), 1.31 (CH=CHCH_2_CH_2_), 1.21 (CH_2_CH_3_). 0.78 (CH_2_CH_3_).

**^13^C{^1^H} NMR** (CDCl_3_, 126 MHz): δ_c_ 132.1-126.0 (HC=CH), 63.4 (CH_2_-OH), 34.0, 32.7, 30.0 (CH_2_CH=CH), 30.6 (CH_2_).

**IR** (ATR-FTIR, cm^–1^): ν 2912m (C-H stretch), 1448m(C-C), 1033 (C-O stretch), 964sh (HC bend from internal [1,4-trans] olefin)


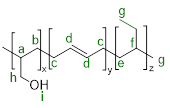

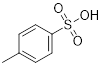


**h**

**i**

Figure S 118. ^1^H NMR spectrum (DMSO-d6, 500 MHz, 298K) of the polyol produced from the hydrogenation of NBR. Table S6– Entry 13.


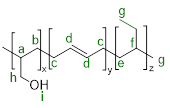


**h**

Figure S 119. ^13^C{^1^H} NMR spectrum (126 MHz, DMSO-d6, 298K) of the polyol produced from the hydrogenation of NBR. Table S6– Entry 13.

Figure S 120. ATR-FT-IR spectrum of the polyol produced from the hydrogenation of NBR. Table S6– Entry 13.

*Table S6 Entry 14*

**^1^H NMR** (CDCl_3_, 500 MHz): δ_H_ 5.35 (HC=CH), 4.32 (OH), 3.27 (H_2_COH), 1.98 (H_2_CH=CH), 1.44 (CHCH_2_OH), 1.33 (CH=CHCH_2_CH_2_), 1.19 (CH_2_CH_3_). 0.78 (CH_2_CH_3_).

**^13^C{^1^H} NMR** (CDCl_3_, 126 MHz): δ_c_ 132.1-128.5 (HC=CH), 63.4 (CH_2_-OH), 34.0, 32.8, 29.7 (CH_2_CH=CH), 30.0 (CH_2_).

**IR** (ATR-FTIR, cm^–1^): ν 2943m (C-H stretch), 1647S (C=C stretch), 1433m(C-C), 1041 (C-O stretch), 966sh (HC bend from internal [1,4-trans] olefin)
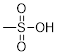
 .


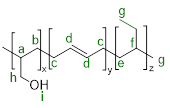


**h**

**i**

Figure S 121. ^1^H NMR spectrum (DMSO-d6, 500 MHz, 298K) of the polyol produced from the hydrogenation of NBR. Table S6– Entry 14.


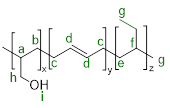


**h**

Figure S 122. ^13^C{^1^H} NMR spectrum (126 MHz, DMSO-d6, 298K) of the polyol produced from the hydrogenation of NBR. Table S6– Entry 14.

Figure S 123. ATR-FT-IR spectrum of the polyol produced from the hydrogenation of NBR. Table S6– Entry 14.

*Table S6 Entry 15*

**^1^H NMR** (CDCl_3_, 500 MHz): δ_H_ 5.53-5.37 (HC=CH), 4.33 (OH), 3.28 (H_2_COH), 2.72 (HCCN), 2.24 (H_2_CH=CH), 2.0 (H_2_CH=CH), 1.57 (CH_2_CHCN) 1.41 (CHCH_2_OH), 1.25 (CH=CHCH_2_CH_2_), 0.83 (CH_2_CH_3_).

**^13^C{^1^H} NMR** (CDCl_3_, 126 MHz): δ_c_ 134.8-125.4 (HC=CH), 122.4 (CN), 63.4 (CH_2_-OH), 34.7, 34.0 (CH_2_CH=CH), 31.2 (CHCH_2_), 29.7 (CHCH_3_)

**IR** (ATR-FTIR, cm^–1^): ν 2916m (C-H stretch), 2237 (C≡N), 1639S (C=C stretch), 1450m(C-C), 1047 (C-O stretch), 966sh (HC bend from internal [1,4-trans] olefin).


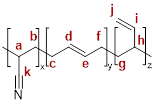

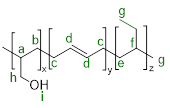

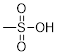


**a**

**h**

**i**

Figure S 124. ^1^H NMR spectrum (DMSO-d6, 500 MHz, 298K) of the polyol produced from the hydrogenation of NBR. Table S6– Entry 15.


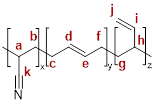

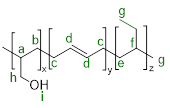


**h**

**k**

Figure S 125. ^13^C{^1^H} NMR spectrum (126 MHz, DMSO-d6, 298K) of the polyol produced from the hydrogenation of NBR. Table S6– Entry 15.

Figure S 126. ATR-FT-IR spectrum of the polyol produced from the hydrogenation of NBR. Table S6– Entry 15.

*Table S6 Entry 16*

**^1^H NMR** (CDCl_3_, 500 MHz): δ_H_ 5.55-5.38 (HC=CH), 5.06 (H_2_C=CH), 2.79 (CHC≡N), 2.28 (H_2_CH=CH), 2.04 (CHCHC=CH_2_)_,_ 1.59 (CH_2_CHC≡N), (CH_2_CH_3_) 1.24, (CH_2_CH_3_) 0.85.

**^13^C{^1^H} NMR** (CDCl_3_, 126 MHz): δ_c_ 133.9-126.0 (HC=CH), 122.4 (C≡N), 34.6, 34.0, 30.9 (CH_2_CH=CH), 32.3 (CHCH_2_), 29.7 (CHCH_3_)

**IR** (ATR-FTIR, cm^–1^): ν 2933m (C-H stretch), 2239sh (C≡N), 1440m(C-C), 968sh (C=C), 914s (HC bend from internal [1,4-trans] olefin).

**a**

Figure S 127. ^1^H NMR spectrum (DMSO-d6, 500 MHz, 298K) of the polyol produced from the hydrogenation of NBR. Table S6– Entry 16.

**k**

Figure S 128. ^13^C{^1^H} NMR spectrum (126 MHz, DMSO-d6) of the polyol produced from the hydrogenation of NBR. Table S6– Entry 16.

Figure S 129. ATR-FT-IR spectrum of the polyol produced from the hydrogenation of NBR. Table S6– Entry 16.

*Table S6 Entry 17*

**^1^H NMR** (CDCl_3_, 500 MHz): δ_H_ 5.53-5.37 (HC=CH), 4.38 (OH), 3.27 (H_2_COH), 2.71 (HCCN), 2.22(H_2_CH=CH), 1.98 (H_2_CH=CH), 1.56 (CH_2_CHCN) 1.41 (CHCH_2_OH), 1.26 (CH=CHCH_2_CH_2_), 0.80 (CH_3_)

**^13^C{^1^H} NMR** (CDCl_3_, 126 MHz): δ_c_ 134.8-125.4 (HC=CH), 122.4 (CN), 63.4 (CH_2_-OH), 34.7, 34.0, 29.7 (CH_2_CH=CH), 31.2 (CHCH_2_).

**IR** (ATR-FTIR, cm^–1^): ν 2920m (C-H stretch), 2237 (C≡N), 1440m(C-C), 1041 (C-O stretch), 970sh (HC bend from internal [1,4-trans] olefin) .

**a**

**a**

**h**

**i**

Figure S 130. ^1^H NMR spectrum (DMSO-d6, 500 MHz, 298K) of the polyol produced from the hydrogenation of NBR. Table S6– Entry 17.

THF

THF

**h**

**k**

Figure S 131. ^13^C{^1^H} NMR spectrum (126 MHz, DMSO-d6, 298K) of the polyol produced from the hydrogenation of NBR. Table S6– Entry 17.

Figure S 132. ATR-FT-IR spectrum of the polyol produced from the hydrogenation of NBR. Table S6– Entry 17.

*Table S6 Entry 18*

**^1^H NMR** (CDCl_3_, 500 MHz): δ_H_ 5.54-5.37 (HC=CH), 5.03-4.95 (H_2_C=CH), 4.36 (OH), 3.27 (H_2_COH), 2.71 (HCCN), 2.23(H_2_CH=CH), 1.99 (H_2_CH=CH), 1.55 (CH_2_CHCN) 1.44 (CHCH_2_OH), 1.32 (CH=CHCH_2_CH_2_), 0.80 (CH_3_)

**^13^C{^1^H} NMR** (CDCl_3_, 126 MHz): δ_c_ 134.8-125.4 (HC=CH), 122.4 (C≡N), 63.4 (CH_2_-OH), 34.7, 34.0, 29.7 (CH_2_CH=CH), 32.7 (CHCH_2_).

**IR** (ATR-FTIR, cm^–1^): ν 2914m (C-H stretch), 2241 (C≡N), 1433m(C-C), 1047 (C-O stretch), 970sh (HC bend from internal [1,4-trans] olefin).

**a**

**h**

**i**

Figure S 133. ^1^H NMR spectrum (DMSO-d6, 500 MHz, 298K) of the polyol produced from the hydrogenation of NBR. Table S6– Entry 18.

THF

THF

**h**

**k**

Figure S 134. ^13^C{^1^H} NMR spectrum (126 MHz, DMSO-d6, 298K) of the polyol produced from the hydrogenation of NBR. Table S6– Entry 18.

Figure S 135. ATR-FT-IR spectrum of the polyol produced from the hydrogenation of NBR. Table S6– Entry 18.

*Table S6 Entry 19*

**^1^H NMR** (CDCl_3_, 500 MHz): δ_H_ 5.34 (HC=CH), 4.32 (OH), 3.27 (H_2_COH), 1.99 (H_2_CH=CH), 1.42 (CHCH_2_OH), 1.31 (CH=CHCH_2_CH_2_), 1.24 (CH_2_CH_3_). 0.86 (CH_2_CH_3_).

**^13^C{^1^H} NMR** (CDCl_3_, 126 MHz): δ_c_ 131.6-128.0 (HC=CH), 63.1 (CH_2_-OH), 33.6, 32.3, 30.0 (CH_2_CH=CH), 30.7 (CH_2_).

**IR** (ATR-FTIR, cm^–1^): ν 2916m (C-H stretch), 1575S and 1539 (C=C stretch), 1469m(C-C), 1033 (C-O stretch), 966sh (C=C).

**i**

**h**

Figure S 136. ^1^H NMR spectrum (DMSO-d6, 500 MHz, 298K) of the polyol produced from the hydrogenation of NBR. Table S6– Entry 19.

Figure S 137. ATR-FT-IR spectrum of the polyol produced from the hydrogenation of NBR. Table S6– Entry 19.

# **S7 Kinetic studies of Nitrile consumption into alcohol**

Figure S 138. Comsumption of Nitrile over time and alcohol yield under the conditions: NBR (0.94 mmol/100 mg), Ru-1 (1%), Triphos 2%, PTSA (15%), THF (1.5 mL), H_2_O (0.5 mL), 150 °C and 40 bar H_2_. Each entry corresponds to a separate reaction that was done and characterized as presented in section S1.

*0 hours*

**^1^H NMR** (CDCl_3_, 500 MHz): δ_H_ 5.55-5.40 (HC=CH), 5.05 (H_2_C=CH), 2.79 (CHC≡N), 2.27 (H_2_CH=CH), 2.05 (CHCHC=CH_2_)_,_ 1.60 (CH_2_CHC≡N),1.24.

**IR** (ATR-FTIR, cm^–1^): ν 2929m (C-H stretch), 2239sh (C≡N), 1435m(C-C), 968sh (HC bend from internal [1,4-trans] olefin), 925s (HC bend from 1,2 vinyl olefin).

**h**

**e**

**b**

**f**

**c f**

**a**

**d**

N

x

y

z

**a**

**b**

**c**

**d**

**d**

**c**

**e**

**f**

**g**

**h**

Figure S 139. ^1^H NMR spectrum (DMSO-d6, 500 MHz, 298K) of commercial NBR.

Figure S 140. ATR-FT-IR spectrum of Commercial Nitrile Butadiene Rubber.

*2-hour reaction*

**^1^H NMR** (CDCl_3_, 500 MHz): δ_H_ 5.54-5.37 (HC=CH), 5.03 (H_2_C=CH), 4.32 (OH), 3.27 (H_2_COH), 2.75 (HCCN), 2.23 (H_2_CH=CH), 2.0 (H_2_CH=CH), 1.57 (CH_2_CHCN) 1.42 (CHCH_2_OH), 1.32 (CH=CHCH_2_CH_2_), 0.85 (CH_3_).

**IR** (ATR-FTIR, cm^–1^): ν 2918m (C-H stretch), 2237 (CN), 1435m(C-C), 1033 (C-O stretch), 968sh (HC bend from internal [1,4-trans] olefin).

**h**

**h**

**a**

**i**

Figure S 141. ^1^H NMR spectrum (DMSO-d6, 500 MHz, 298K) of the polyol produced from the hydrogenation of NBR obtained after 2 hours.

Figure S 142. ATR-FT-IR spectrum of the polyol produced from the hydrogenation of NBR obtained after 2 hours.

*3-hour reaction*

**^1^H NMR** (CDCl_3_, 500 MHz): δ_H_ 5.53-5.35 (HC=CH), 5.03 (H_2_C=CH), 4.31 (OH), 3.27 (H_2_COH), 2.71 (HCCN), 2.21 (H_2_CH=CH), 1.98 (H_2_CH=CH), 1.54 (CH_2_CHCN) 1.40 (CHCH_2_OH), 1.31 (CH=CHCH_2_CH_2_), 0.82 (CH_3_).

**IR** (ATR-FTIR, cm^–1^): ν 2918m (C-H stretch), 2235 (CN), 1647 (C=C stretch), 1435m(C-C), 1033 (C-O stretch), 970sh (HC bend from internal [1,4-trans] olefin).

**h**

**a**

**h**

**i**

Figure S 143. ^1^H NMR spectrum (DMSO-d6, 500 MHz, 298K) of the polyol produced from the hydrogenation of NBR obtained after 3 hours.

Figure S 144. ATR-FT-IR spectrum of the polyol produced from the hydrogenation of NBR obtained after 3 hours.

*6-hour reaction*

**^1^H NMR** (CDCl_3_, 500 MHz): δ_H_ 5.52-5.36 (HC=CH), 5.03 (H_2_C=CH), 4.32 (OH), 3.27 (H_2_COH), 2.73 (HCCN), 2.21 (H_2_CH=CH), 1.99 (H_2_CH=CH), 1.57 (CH_2_CHCN) 1.43 (CHCH_2_OH), 1.32 (CH=CHCH_2_CH_2_), 0.85 (CH_3_).

**IR** (ATR-FTIR, cm^–1^): ν 2918m (C-H stretch), 2241 (CN), 1435m(C-C), 1035 (C-O stretch), 968sh (HC bend from internal [1,4-trans] olefin).

**h**

**h**

**a**

**i**

Figure S 145. ^1^H NMR spectrum (DMSO-d6, 500 MHz, 298K) of the polyol produced from the hydrogenation of NBR obtained after 6 hours.

Figure S 146. ATR-FT-IR spectrum of the polyol produced from the hydrogenation of NBR obtained after 6 hours.

*14-hour reaction*

**^1^H NMR** (CDCl_3_, 500 MHz): δ_H_ 5.34 (HC=CH), 4.32 (OH), 3.27 (H_2_COH), 2.67 (HCCN), 1.98 (H_2_CH=CH), 1.40 (CHCH_2_OH), 1.32 (CH=CHCH_2_CH_2_), 0.85 (CH_3_).

**IR** (ATR-FTIR, cm^–1^): ν 2916m (C-H stretch), 1433m(C-C), 1033 (C-O stretch), 966sh (HC bend from internal [1,4-trans] olefin).

**a**

**h**

**i**

Figure S 147. ^1^H NMR spectrum (DMSO-d6, 500 MHz, 298K) of the polyol produced from the hydrogenation of NBR obtained after 14 hours.

Figure S 148. ATR-FT-IR spectrum of the polyol produced from the hydrogenation of NBR obtained after 14 hours.

*20-hour reaction*

**^1^H NMR** (CDCl_3_, 500 MHz): δ_H_ 5.34 (HC=CH), 4.35 (OH), 3.26 (H_2_COH), 1.98 (H_2_CH=CH), 1.38 (CHCH_2_OH), 1.30 (CH=CHCH_2_CH_2_), 1.22 (CH_2_CH_3_). 0.78 (CH_2_CH_3_).

**IR** (ATR-FTIR, cm^–1^): ν 2916m (C-H stretch), 1433m(C-C), 1033 (C-O stretch), 966sh (HC bend from internal [1,4-trans] olefin).

**h**

**i**

Figure S 149. ^1^H NMR spectrum (DMSO-d6, 500 MHz, 298K) of the polyol produced from the hydrogenation of NBR obtained after 20 hours.

Figure S 150. ATR-FT-IR spectrum of the polyol produced from the hydrogenation of NBR obtained after 20 hours.

# **S8 Catalytic hydrogenation of NBR end-of-life products**

## *Starting Material characterization*

Gray Gloves

Figure S 151. ATR-FT-IR spectrum of gray gloves (post-consumer NBR waste).

Figure S 152. TGA of gray gloves (post-consumer NBR waste). Onset temperature of 419 °C.

Olefins (C=C)

Nitrile (C≡N)

(C-C)

Figure S 153.^13^C (9.4 T, 12.5 kHz CP MAS) NMR spectrum of gray gloves (post-consumer NBR waste).^[5]^

Figure S 154. DSC of gray glove waste. Amorphous material with glass transition temperature of -29.2 °C.

**1**

Figure S 155 GPC of gray glove waste. (1) M_n_ : 12923 Da PDI: 6.49.

Table S 7. Elemental analysis of gray gloves.
C,75.76; H,8.72; N, 6.45; S, 0.88

Figure S 156**.** ATR-FT-IR spectrum of blue gloves (post-consumer NBR waste).

Figure S 157. TGA of blue gloves (post-consumer NBR waste). Onset temperature of 412 °C.

(C-C)

Olefins (C=C)

Nitrile (C≡N)

Figure S 158. ^13^C (9.4 T, 12.5 kHz CP MAS) NMR spectrum of blue gloves (post-consumer NBR waste).^[5]^

Figure S 159.DSC of blue glove waste. Amorphous material with glass transition temperature of -14.9 °C.

**1**

Figure S 160. GPC of blue glove waste. (1) M_n_ : 11117 Da, PDI: 4.26.

Figure S 161. ATR-FT-IR spectrum of O-Ring (post-consumer NBR waste). We speculate that the poor baseline is due to the compact nature of the material and its black color. The material was cut as thin as possible using scissors and subjected to the IR. Thicker material showed poor transmittance.

Figure S 162. TGA of O-Ring (post-consumer NBR waste).Onset temperature of 462 °C.

(C-C)

Olefins (C=C)

Nitrile (C≡N)

Figure S 163. ^13^C (9.4 T, 12.5 kHz CP MAS) NMR spectrum of O-ring (post-consumer NBR waste). ^[5]^

Figure S 164. DSC of gray glove waste. Amorphous material with a glass transition temperature of -41.0 °C.

**1**

Figure S 165. GPC of O ring (post-consumer NBR waste). (1) M_n_ : 10484 Da, PDI: 4.64.

## *Devulcanization procedure*

Devulcanisation was carried out using a method adapted from the literature.^[6]^ 100 mg of ground end-of-life NBR product was placed in a 10 mL Schlenk flask along with 1 mL of nitrobenzene. The flask was sealed and heated in an oil bath at 180 °C for 16 hours under vigorous stirring. After the reaction, the resulting black homogeneous solution was removed from the flask by washing with equal parts of THF. The combined solution was filtered, and the filtrate was concentrated using a rotavapor. The residue was then washed with water (to separate butylated hydroxytoluene), and the organic layer was precipitated with hexane to yield a black rubbery material. This material was washed three times with hexane and dried under vacuum overnight. The purified product was subsequently characterized by NMR and IR spectroscopy and then subjected to hydrogenation.

Hexane

180°C

Figure S 166. Devulcanization procedure for end-of-life materials.

*Additives recovered*

Figure S 167 GC-MS of yellow powder extracted from gloves (identified as Butylated hydroxytoluene or BHT).

Figure S 168 FT-IR of yellow powder extracted from gloves.

*Devulcanized end-of-life materials- gray gloves*

**^1^H NMR** (CDCl_3_, 500 MHz): δ_H_ 5.55-5.39 (HC=CH), 5.03 (H_2_C=CH), 2.77 (CHC≡N), 2.24 (H_2_CH=CH), 2.04 (CHCHC=CH_2_)_,_ 1.57 (CH_2_CHC≡N),1.25.

**IR** (ATR-FTIR, cm^–1^): ν 2927m (C-H stretch), 2238sh (C≡N), 1521 (C=C stretch) 1346m(C-C), 966sh (HC bend from internal [1,4-trans] olefin), 929s (HC bend from 1,2 vinyl olefin).

Figure S 169. ATR-FT-IR spectrum of devulcanized gray gloves.

**b**

**f**

**c f**

**a**

**h**

**d,g**

N

x

y

z

**a**

**b**

**c**

**d**

**d**

**c**

**e**

**f**

**g**

**h**

Figure S 170. ^1^H NMR spectrum (CDCl_3_, 500 MHz, 298K) of devulcanized gray gloves.

**f**

**a**

**f**

**c**

**k**

**d e**

Figure S 171. ^13^C{^1^H} NMR spectrum (126 MHz, CDCl_3_, 298K) of the devulcanized material obtained from gray gloves.

**k**

Figure S 172. ^1^H, ^13^C- HSQC NMR (CDCl_3_) spectrum of the devulcanized material obtained from gray gloves.

N

x

y

z

**a**

**b**

**c**

**d**

**d**

**c**

**e**

**f**

**g**

**h**

**c f**

**f**

**d,g**

**h**

**a**

**b**

**b**

**f**

**c f**

**h**

**d,g**

**a**

Figure S 173. ^1^H NMR spectrum (CDCl_3_, 500 MHz, 298K) of devulcanized gray gloves (Blue) and NBR virgin (Red). Ratio of 1:0.28 Butadiene:Acrylonitrile.

Figure S 174. ATR-FT-IR spectrum of devulcanized gray gloves, gray gloves and NBR.

**1**

Figure S 175. GPC of devulcanized gray gloves in DMF. (1) M_n_ = 12923 Da, PDI: 6.49.

Figure S 176. TGA of devulcanized gray gloves. Onset temperature of 407°C.

Figure S 177. DSC of gray glove waste devulcanized. Amorphous material with glass transition temperature of -20.7 °C.

Figure S 178. ^13^C (14.1 T, 12.5 kHz CP MAS) NMR spectrum of devulcanized gray gloves (post-consumer NBR waste)

Table S 8. Elemental analysis of devulcanized gray gloves. Higher O contents due to leftover Nitrobenzene.

C,75.00; H,8.09; N, 6.81; S, 0.61, O, 4.83.

Table S 9. ICP-MS of devulcanized gray gloves for detection of Ruthenium

*Devulcanized blue gloves*

**^1^H NMR** (CDCl_3_, 500 MHz): δ_H_ 5.54 (HC=CH), 5.03 (H_2_C=CH), 2.75 (CHC≡N), 2.23 (H_2_CH=CH), 2.0 (CHCHC=CH_2_)_,_ 1.56 (CH_2_CHC≡N),1.25, 0.82 (CH_3_)

**IR** (ATR-FTIR, cm^–1^): ν 2927m (C-H stretch), 2237sh (C≡N), 1523 (C=C stretch) 1346m(C-C), 968sh (HC bend from internal [1,4-trans] olefin), 914s (HC bend from 1,2 vinyl olefin).

**d,g**

**f**

**b**

**c**

**a**

**h**

N

x

y

z

**a**

**b**

**c**

**d**

**d**

**c**

**e**

**f**

**g**

**h**

Figure S 179. ^1^H NMR spectrum (DMSO-d6, 500 MHz, 298K) of devulcanized blue gloves.

**f**

**a**

**c**

**k**

**d e**

Figure S 180. ^13^C{^1^H} NMR spectrum (126 MHz, CDCl_3_, 298K) of the devulcanized material obtained from blue gloves.

Figure S 181. ^1^H, ^13^C- HSQC NMR (CDCl_3_) spectrum of the devulcanized material obtained from gray gloves.

Figure S 182. ^1^H NMR spectrum (CDCl_3_, 500 MHz, 298K) of devulcanized blue gloves (Blue) and NBR virgin (Red).

Figure S 183. ATR-FT-IR spectrum of devulcanized blue gloves.

Figure S 184. ATR-FT-IR spectrum of devulcanized blue gloves, blue gloves and virgin NBR.

Figure S 185. TGA of devulcanized blue gloves. Onset temperature of 414 °C.

Figure S 186. DSC of blue glove waste devulcanized. Amorphous material with glass transition temperature of -11.3 °C.

**1**

Figure S 187 GPC of devulcanized blue glove waste in DMF. (1) M_n_ = 11117 Da, PDI: 4.27.

*O-ring*

**^1^H NMR** (CDCl_3_, 500 MHz): δ_H_ 5.52-5.37 (HC=CH), 5.05 (H_2_C=CH), 2.74 (CHC≡N), 2.23 (H_2_CH=CH), 2.0 (CHCHC=CH_2_)_,_ 1.56 (CH_2_CHC≡N),1.25.

**IR** (ATR-FTIR, cm^–1^): ν 2905m (C-H stretch), 2238sh (C≡N), 1346m(C-C), 953sh (HC bend from internal [1,4-trans] olefin), 929s (HC bend from 1,2 vinyl olefin).

**c**

**f**

**b**

**a**

**d,g**

**h**

N

x

y

z

**a**

**b**

**c**

**d**

**d**

**c**

**e**

**f**

**g**

**h**

Figure S 188. ^1^H NMR spectrum (Acetone-d6, 500 MHz, 298K) of devulcanized O- Ring.

Figure S 189. ^1^H NMR spectrum (Acetone-d6, 500 MHz, 298K) of devulcanized O ring (Blue) and NBR virgin (Red).

Figure S 190. ^1^H, ^13^C- HSQC NMR (Acetone-d6) spectrum of the devulcanized material obtained from O rings.

Figure S 191.TGA of devulcanized O rings. Onset temperature of 411 °C.

Figure S 192. DSC of O ring waste devulcanized. Amorphous material with glass transition temperature of -17.7 °C.

**1**

Figure S 193. GPC of devulcanized O rings in DMF. (1) M_n_ = 10484 Da, PDI: 4.64.

Figure S 194. ^13^C (14.1 T, 12.5 kHz CP MAS) NMR spectrum of devulcanized O-Ring (post-consumer NBR waste)

## *End-of-life materials post-hydrogenation*

Table S 10 Hydrogenation of end-of-life materials.

Table S10-Entry 1

**^1^H NMR** (CDCl_3_, 500 MHz): δ_H_ 5.59 (HC=CH), 4.99 (HC=CH), 2.60 (H_2_CNH_2_), 2.05 (H_2_CH=CH), 1.43 (CHCH_2_NH_2_ or CHCH=CH_2_), 1.36 (CHCH_2_).

**k**

Figure S 195. ^1^H NMR spectrum (CDCl_3_, 500 MHz, 298K) of the polyamine produced from the hydrogenation of gray gloves. Table S10 – Entry 1

**k**

Figure S 196. ^1^H, ^13^C- HSQC NMR (CDCl_3_) spectrum of the devulcanized material obtained from O rings.

Figure S 197. ATR-FT-IR spectrum of polyamine obtained from the hydrogenation of gray gloves

Figure S 198. ATR-FT-IR spectrum of polyamine obtained from the hydrogenation of gray gloves and comparison with devulcanized gray gloves and NBR.

Figure S 199. TGA of polyamine obtained from hydrogenation of gray gloves. Onset temperature of 415 °C

Figure S 200. DSC of polyamine obtained from the hydrogenation of gray gloves. Amorphous material with glass transition temperature of -22.3 °C.

Table S 11. Elemental analysis of polyamine obtained from the hydrogenation of gray gloves.

C,69.01; H,8.30; N, 5.87.

Table S 12. ICP-MS analysis of polyamine obtained from hydrogenation for detection of Ruthenium.

*Table S10 – Entry 2*

**^1^H NMR** (CDCl_3_, 500 MHz): δ_H_ 5.53 (HC=CH), 4.32 (OH), 3.26 (H_2_COH), 1.98 (H_2_CH=CH), 1.42 (CHCH_2_OH), 1.31 (CH=CHCH_2_CH_2_), 1.21 (CH_2_CH_3_). 0.78 (CH_2_CH_3_).

**IR** (ATR-FTIR, cm^–1^): ν 2920m (C-H stretch), 1433m(C-C), 1033 (C-O stretch), 968sh (C=C).

**i**

**h**

Figure S 201. ^1^H NMR spectrum (DMSO-d6, 500 MHz, 298K) of the polyol produced from the hydrogenation of gray gloves. Table S10 – Entry 2

Figure S 202. ^13^C{^1^H} NMR spectrum (126 MHz, DMSO, 298K) of the polyol produced from the hydrogenation of gray gloves. Table S10 – Entry 2

Figure S 203. ^1^H, ^13^C- HSQC NMR (DMSO-d6) spectrum of the polyol produced from the hydrogenation of gray gloves. Table S7 – Entry 2

Figure S 204**.** ATR-FT-IR spectrum of the polyol produced from the hydrogenation of gray gloves. Table S10- Entry 2.

Figure S 205. ATR-FT-IR spectrum of the polyol produced from the hydrogenation of gray gloves. Table S10- Entry 2.

Figure S 206. TGA of the polyol produced from the hydrogenation of gray gloves. Onset temperature of 386°C

**1**

Figure S 207. GPC of polyol obtained from the hydrogenation of devulcanized gray gloves in DMF (Table S10. Entry 2). (1) M_n_ = 12 064 Da, PDI: 7.13.

Figure S 208. DSC of polyol obtained from the hydrogenation of gray gloves. Amorphous material with glass transition temperature of -0.9 °C.

Table S 13. Elemental analysis of polyol obtained from the hydrogenation of gray gloves.

C,71.48; H,8.54; N, 1.98; O, 12.44.

Table S 14. ICP-MS of polyol obtained from the hydrogenation of gray gloves for detection of Ruthenium

*Table S10 – Entry 3*

**^1^H NMR** (CDCl_3_, 500 MHz): δ_H_ 5.40 (HC=CH), 2.58 (H_2_CNH_2_), 2.03 (H_2_CH=CH), 1.41 (CHCH_2_NH_2_ or CHCH=CH_2_), 0.88 (CH_2_CH_3_).

**k**

Figure S 209. ^1^H NMR spectrum (CDCl3, 500 MHz, 298K) of the polyamine produced from the hydrogenation of O-Ring. Table S10 – Entry 3

Figure S 210. ^1^H, ^13^C- HSQC NMR (CDCl_3_) spectrum of the polyamine produced from the hydrogenation of O-Ring. Table S10 – Entry 3

Figure S 211. TGA of the polyamine produced from the hydrogenation of O-Ring. Onset temperature of 412°C

Figure S 212. DSC of polyamine obtained from the hydrogenation of O-Ring. Amorphous material with glass transition temperature of -12.5 °C.

*Table S10– Entry 4*

**^1^H NMR** (CDCl_3_, 500 MHz): δ_H_ 5.34 (HC=CH), 4.32 (OH), 3.26 (H_2_COH), 1.97 (H_2_CH=CH), 1.43 (CHCH_2_OH), 1.35 (CH=CHCH_2_CH_2_), 1.21 (CH_2_CH_3_). 0.85 (CH_2_CH_3_).

**h**

**i**

Figure S 213. ^1^H NMR spectrum (DMSO-d6, 500 MHz, 298K) of the polyol produced from the hydrogenation of O-ring. Table S10– Entry 4

Figure S 214. ^13^C{^1^H} NMR spectrum (126 MHz, DMSO-d6, 298K) of the polyol produced from the hydrogenation of O-ring. Table S10 – Entry 4

Figure S 215. ^1^H, ^13^C- HSQC NMR (DMSO-d6) spectrum of the polyol produced from the hydrogenation of O-Ring. Table S10 – Entry 4

Figure S 216. ATR-FT-IR spectrum of the polyol produced from the hydrogenation of O-Ring. Table S10- Entry 4.

Figure S 217. TGA of the polyol produced from the hydrogenation of O-Ring. Onset temperature of 327°C

**1**

Figure S 218. GPC of polyol obtained from the hydrogenation of devulcanized O-Ring in DMF (Table S7. Entry 4). (1) M_n_ = 9251 Da, PDI: 6.22

Figure S 219. DSC of polyol obtained from the hydrogenation of O-Ring. Amorphous material with glass transition temperature of 6.8 °C.

*Table S10 – Entry 5*

**k**

Figure S 220**.** ^1^H NMR spectrum (CDCl_3_, 500 MHz, 298K) of the polyamine produced from the hydrogenation of blue gloves. Table S10 – Entry 5.

**k**

Figure S 221. HSQC NMR (CDCl_3_) spectrum of the polyol produced from the hydrogenation of blue gloves. Table S10 – Entry 5

Figure S 222. ATR-FT-IR spectrum of the polyamine produced from the hydrogenation of blue gloves Table S10- Entry 5.

Figure S 223. ATR-FT-IR spectrum of the polyamine produced from the hydrogenation of blue gloves, Table S10- Entry 5.

Figure S 224. TGA of the polyol produced from the hydrogenation of the blue glove. Onset temperature of 417°C

Figure S 225. DSC of polyamine obtained from the hydrogenation of blue gloves. Amorphous material with glass transition temperature of -14.2 °C.

*Table S10 – Entry 6*

**^1^H NMR** (CDCl_3_, 500 MHz): δ_H_ 5.34 (HC=CH), 4.36 (OH), 3.26 (H_2_COH), 1.98 (H_2_CH=CH), 1.41 (CHCH_2_OH), 1.33 (CH=CHCH_2_CH_2_), 1.21 (CH_2_CH_3_). 0.82 (CH_2_CH_3_).

**h**

**i**

Figure S 226**.** ^1^H NMR spectrum (DMSO-d6, 500 MHz, 298K) of the polyol produced from the hydrogenation of blue gloves. Table S10 – Entry 6.

Figure S 227. ^13^C{^1^H} NMR spectrum (126 MHz, DMSO-d6, 298K) of the polyol produced from the hydrogenation of blue gloves. Table S10 – Entry 6.

**h**

Figure S 228. HSQC NMR (DMSO-d6) spectrum of the polyol produced from the hydrogenation of blue gloves. Table S10 – Entry 6

Figure S 229. ATR-FT-IR spectrum of the polyol produced from the hydrogenation of blue gloves Table S10- Entry 6.

Figure S 230. ATR-FT-IR spectrum of the polyol produced from the hydrogenation of blue gloves, Table S10- Entry 6.

Figure S 231. TGA of the polyol produced from the hydrogenation of blue gloves. Onset temperature of 401°C

Figure S 232. DSC of polyol obtained from the hydrogenation of blue gloves. Amorphous material with a glass transition temperature of -11.5 °C.

**1**

Figure S 233. GPC of polyol obtained from the hydrogenation of devulcanized blue ring in DMF (Table S10, Entry 6). (1) M_n_ = 9881 Da, PDI: 3.51

# **S9 Small molecule experiments**

**(8)**

Figure S 234. Small molecule tests for mechanistic studies.

*Experiment 1*

**^1^H NMR** (CDCl_3_, 500 MHz): δ_H_ 5.55-5.39 (HC=CH), 5.08 (H_2_C=CH), 2.79 (CHC≡N), 2.29 (H_2_CH=CH), 2.05 (CHCHC=CH_2_)_,_ 1.58 (CH_2_CHC≡N),1.24, 0.87 (CH_2_CH_3_)

**^13^C{^1^H} NMR** (CDCl_3_, 126 MHz): δ_c_ 132.69-125.97 (HC=CH), 122.43 (CN), 34.68, 30.93 (CH_2_CH=CH), 29.87 (CHCH_3_)

**h**

**e**

**b**

**f**

**c**

**a**

**d,g**

N

x

y

z

**a**

**b**

**c**

**d**

**d**

**c**

**e**

**f**

**g**

**h**

N

x

y

z

**a**

**b**

**c**

**d**

**d**

**c**

**e**

**f**

**g**

**h**

Figure S 235. ^1^H NMR spectrum (DMSO-d6, 500 MHz, 298K) of mechanistic study 1.

**k**

Figure S 236. ^13^C{^1^H} NMR spectrum (126 MHz, DMSO-d6) of mechanistic study 1.

*Experiment 2*

**^1^H NMR** (CDCl_3_, 500 MHz): δ_H_ 5.57-5.43 (HC=CH), 5.07 (H_2_C=CH), 2.81 (CHC≡N), 2.30 (H_2_CH=CH), 2.05 (CHCHC=CH_2_)_,_ 1.61 (CH_2_CHC≡N),1.24, 0.85 (CH_2_CH_3_)

**^13^C{^1^H} NMR** (CDCl_3_, 126 MHz): δ_c_ 133.90-125.97 (HC=CH), 122.41 (CN), 34.68, 32.34, 29.66 (CH_2_CH=CH), 31.22 (CHCH_2_).

**h**

**a**

Figure S 237. ^1^H NMR spectrum (DMSO-d6, 500 MHz, 298K) of mechanistic study 2.

**k**

Figure S 238. ^13^C{^1^H} NMR spectrum (126 MHz, DMSO-d6) of mechanistic study 2.

*Experiment 3*

See Table 2-Entry 9

*Experiment 4A*

**^1^H NMR** (CDCl_3_, 500 MHz): δ_H_ 2.64 (CH_2_NH_2_), 1.42 (CH_2_CH_2_NH_2_), 1.31 (CH_2_CH_2_CH_2_)

Figure S 239. GC-MS chromatogram of mechanistic study 4A.

Mesitylene

Mesitylene

IPA

IPA

**b**

**c**

**c**

**c**

**b**

**b**

**a**

**a**

**a**

Figure S 240. ^1^H NMR spectrum (CDCl_3_, 500 MHz, 298K) of mechanistic study 4A.

*Experiment 4B*

**^1^H NMR** (CDCl_3_, 500 MHz): δ_H_ 3.64 (CH_2_OH), 1.58 (CH_2_CH_2_OH), 1.40 (CH_2_CH_2_CH_2_)

Figure S 241. GC-MS chromatogram of mechanistic study 4B.

Mesitylene

0.5 mmol

3H

**a**

**a**

**b**

**c**

**c**

**b**

**c**

**b**

THF

**a**

DCM

THF

Figure S 242. ^1^H NMR spectrum (CDCl_3_, 500 MHz, 298K) of mechanistic study 4B.

*Experiment 5*

**^1^H NMR** (CDCl_3_, 500 MHz): δ_H_ 3.62 (CH_2_OH), 1.54 (CH_2_CH_2_OH), 1.24 (CH_2_CH_2_CH_2_). 0.85 (CH_2_CH_3_)

Mesitylene

Figure S 243. GC-MS chromatogram of mechanistic study 5.

THF

**d**

**c**

**b**

THF

Mesitylene

0.065 mmol

6H

DCM

Mesitylene

0.065 mmol

3H

**a**

**a**

**c**

**c**

**c**

**c**

**c**

**c**

**d**

**b**

**a**

Figure S 244. ^1^H NMR spectrum (CDCl_3_, 500 MHz, 298K) of mechanistic study 5.

*Experiment 6*

**^1^H NMR** (CDCl_3_, 500 MHz): δ_H_ 7.38-7.26 (Ar. CH=CH), 5.83 (CHOH), 2.50 (OH).

Mesitylene

Figure S 245. GC-MS chromatogram of mechanistic study 6.

**b**

**b**

**b**

**a**

**c**

**c**

**b**

**b**

**b**

**b**

**b**

**b**

**b**

**b**

**a**

Mesitylene

0.0708 mmol

9H

Mesitylene

0.0708 mmol

3H

THF

THF

THF

THF

Figure S 246. ^1^H NMR spectrum (CDCl_3_, 500 MHz, 298K) of mechanistic study 6.

*Experiment 7*

**^1^H NMR** (CDCl_3_, 500 MHz): δ_H_ 7.80-7.48 (Ar. CH=CH)

**^13^C{^1^H} NMR** (CDCl_3_, 126 MHz): δ_c_ 196.72 (C=O), 137.66 (CHC=O), 132.39 (CH=CH), 130.05 (CH=C), 129.03 (CH=CH)

Figure S 247. GC-MS chromatogram of mechanistic study 7.

**c**

**a**

**b**

**b**

**a**

**a**

**b**

**c**

**b**

**a**

**c**

**b**

**a**

Figure S 248. ^1^H NMR spectrum (CDCl_3_, 500 MHz, 298K) of mechanistic study 7.

**b**

**c**

**d**

**e**

**e**

**e**

**e**

**e**

**d**

**d**

**d**

**d**

**c**

**c**

**a**

**b**

**b**

**a**

Figure S 249. ^13^C{^1^H} NMR spectrum (126 MHz, CDCl_3_) of mechanistic study 7.

*Experiment 8*

e

a,c,f

h

g

b,d

Mesitylene

0.14 mmol

3H

Figure S 250. ^1^H NMR spectrum (CDCl_3_, 500 MHz, 298K) of 20 mg (0.37 mmol) of Polybutadiene (M_n_ 5000 g/mol).

$$Mmol of internal double bond in PBD= \frac{\int double bond CH}{\int Mesitylene CH}*\frac{3 (comes from no of protons in mesitylene)}{2 (comes from no of protons in double bonds)}*mmol of mesityelene$$

$$Mmol of internal double bond in Polyol= \frac{2.0}{1.33}*\frac{3}{2}*0.14 mmol$$

$$Mmol of internal double bond in Polyol=0.32 mmol$$

$$Mmol of vinyl double bond in Polyol=0.045 mmol$$

IPA

THF

THF

IPA

Mesitylene

a,c,f

Mesitylene

0.14 mmol

3H

g

b,d

h

Figure S 251. ^1^H NMR spectrum (CDCl_3_, 500 MHz, 298K) of 108 mg (2 mmol) of product of hydrogenation of Polybutadiene (M_n_ 5 000 g/mol).

$$Mmol of internal double bond in Polyol= \frac{\int double bond CH}{\int Mesitylene CH}*\frac{3 (comes from no of protons in mesitylene)}{2 (comes from no of protons in double bonds)}*mmol of mesityelene$$

$$Mmol of internal double bond in Polyol= \frac{2.0}{0.24}*\frac{3}{2}*0.14 mmol$$

$$Mmol of internal double bond in Polyol=1.75 mmol$$

$$Mmol of vinyl double bond in Polyol=0.22 mmol$$

# **S10 CO_2_ Capture tests**

The CO₂ capture experiment was carried out using a Netzsch STA449F5 (Simultaneous thermal analyzer) under controlled gas and temperature conditions. The sample was first heated dynamically to 120 °C under a constant flow of nitrogen (N₂), followed by an isothermal hold at 120 °C for 1 hour to ensure desorption of moisture and volatile impurities. Subsequently, the sample was cooled dynamically to 90 °C under N₂ and held isothermally at this temperature for 30 minutes. While maintaining 90 °C, the purge gas was switched from N₂ to CO₂, and the sample was exposed to CO₂ for 4 hours to allow adsorption. After the adsorption step, the gas was switched back to N₂, and the sample was heated dynamically to 120 °C to initiate desorption. An isothermal hold at 120 °C for 1 hour under N₂ was then performed before cooling the system down to room temperature.

Figure S 252. CO_2_ capture test for polyamine obtained from NBR hydrogenation.

Figure S 253. CO_2_ capture test for commercial NBR sample.

Figure S 254. CO_2_ capture test for commercial branched PEI sample of M_n_= 10 000 g/mol.

Figure S 255. CO_2_ capture test for commercial linear PEI sample of M_n_=2 100 g/mol.

# **S11 Mechanical properties**

Rectangular specimens of the NBR-based elastomer and the polyol formulation were characterised in tension using the strip-test configuration described in ASTM D882 / ISO 1926. Each sample was clamped in the universal testing machine and stretched at a 50 mm/min strain rate while stress–strain data were recorded up to failure, providing the tensile properties required for further analysis.

# **S12 Economic and environmental assessment**

## S12.1 Life cycle assessment:

The life cycle assessment (LCA) in this study refers to a cradle-to-gate footprint evaluation, focusing on four environmental footprint impact categories: Global warming potential (GWP), acidification, eutrophication, and water consumption. These impact categories are the key metrics provided by the ACS Green Chemistry Institute's streamlined PMI-LCA tool^[7]^ to assess and compare environmental footprints for complex organic reactions.^[8]^

A comparative life cycle assessment (LCA) per kg demonstrates a clear environmental advantage of the catalytic reduction protocol over the conventional LiAlH₄ route. The process mass intensity (PMI) is reduced from 87 to 34, accompanied by a decrease in overall mass intensity (322 to 109 kg input per kg API). The cumulative energy demand is nearly halved (7079 to 3833 MJ, −48%), highlighting the significant upstream and operational complications associated with stoichiometric aluminum hydrides. Most notably, the global warming potential decreases (2531 to 166 kg CO₂ equiv.), underscoring the high embedded carbon footprint of LiAlH₄ production and salt-generating workups.

Beyond carbon emissions, the catalytic process also substantially lowers broader environmental impacts. Acidification and eutrophication potentials are slightly reduced, consistent with the elimination of inorganic aluminum salt waste streams and diminished mineral acid/base neutralization steps, but the use of ruthenium in the system. Collectively, these results demonstrate that replacing stoichiometric LiAlH₄ with catalytic hydrogenation delivers a step-change improvement in material efficiency, energy demand, and environmental footprint, reinforcing the sustainability advantage of catalytic methodologies in modern chemical manufacturing.

Figure S 256. Life cycle assessment of the benchmark procedure in literature with LiAlH_4_^[9]^ and this paper. LiAlH_4_ modelling (based on comparatively high energy consumption for steam and electricity based on pharma industry average data organic synthesis, suitable for organic synthesis, suitable for chemicals with high purity in pharma that are not simple organic chemicals).^[10]^

Figure S 257. Life cycle assessment of the benchmark procedure in literature with LiAlH_4_^[9]^ and this paper. LiAlH_4_ modelling (based on comparatively high energy consumption for steam and electricity based on pharma industry average data organic synthesis, suitable for organic synthesis, suitable for chemicals with high purity in pharma that are not simple organic chemicals).^[10]^

## S12.2 E Factor

The environmental performance of the developed hydrogenation routes was evaluated using the E-factor metric and compared against a representative literature method based on LiAlH₄-mediated hydrogenation of NBR to polyamines (Table S15). The hydrogenation strategy reported in this work exhibits substantially lower E-factors (18.8–34.6 kg waste kg⁻¹ product for polyol and polyamine formation, respectively) than the LiAlH₄-based approach (101 kg waste kg⁻¹ product). This improvement reflects the avoidance of stoichiometric hydride reagents and the associated inorganic salt waste, which dominate the mass balance in conventional reductions. Even when post-consumer NBR waste is used as feedstock, the E-factors obtained in this work (75.3-59.5 kg waste kg⁻¹ product) remain lower than that of the literature benchmark, highlighting the intrinsic sustainability advantage of catalytic hydrogenation routes for NBR valorisation despite the increased complexity of real waste streams.

Table S 15. E factor of the benchmark procedure in the literature and the procedures presented in this paper.

|  | **This work** | |  | **This work** | |  |
| --- | --- | --- | --- | --- | --- | --- |
|  | **NBR hydrogenation to polyol** | **NBR hydrogenation to polyamine** | **NBR Hydrogenation with LiAlH_4_ to polyamine** | **Post consumer waste to polyamine** | **Post consumer waste to polyol** | **Post consumer waste Hydrogenation with LiAlH_4_ to polyamine** |
| E factor (kg waste * (kg product)^-1^ | 18.8 | 34.6 | 101 | 75.3 (Hexane)  46.6 (Kugelrohr)* | 59.5 (Hexane)  30.8 (Kugelrohr)* | 142 (Hexane)  122.2 (Kugelrohr)* |

*Kugelrohr allows for the evaporation of Nitrobenzene at low pressure and isolation of devulcanised NBR without the use of extra solvent.

**For NBR hydrogenation to polyol:**

Waste amount:
Ru(acac)_3_ = 0.0036 g

Triphos = 0.012 g

PTSA = 0.028 g

THF = 1.335 g

H_2_O = 0.500 g

Product amount: 0.105g

**For NBR hydrogenation to polyamine:**

RuMACHO BH = 0.0058 g

KOtBu = 0.0058 g

THF = 2.667 g

IPA = 0.785 g

Product amount: 0.103g

**NBR Hydrogenation with LiAlH_4_ to polyamine.**

THF = 13.35 g

LiAlH_4_ = 1.5 g

Methanol = 35.64 g

Product amount: 0.513 g

**Post consumer waste to polyol:**

Nitrobenzene = 1.20 g

THF = 0.889 g

Hexane = 1.98 g

Ru(acac)_3_ = 0.0036 g

Triphos = 0.012 g

PTSA = 0.028 g

THF = 1.335 g

H_2_O = 0.500 g

Product amount: 0.105g

**Post consumer waste to polyamine:**

Nitrobenzene = 1.20 g

THF = 0.889 g

Hexane = 1.98 g

RuMACHO BH = 0.0058 g

KOtBu = 0.0058 g

THF = 2.667 g

IPA = 0.785 g

Product amount: 0.103g

**Post consumer waste Hydrogenation with LiAlH_4_ to polyamine:**

Nitrobenzene = 6 g

THF = 4.44 g

Hexane = 9.9 g

THF = 13.35 g

LiAlH_4_ = 1.5 g

Methanol = 35.64 g

Product amount: 0.513 g

**S13 References**

[1] M. Peña‐López, P. Piehl, S. Elangovan, H. Neumann, M. Beller, “Manganese‐Catalyzed Hydrogen‐Autotransfer C−C Bond Formation: α‐Alkylation of Ketones with Primary Alcohols” *Angewandte Chemie International Edition* **2016**, *55*, 14967–14971.

[2] T. Yasin, S. Ahmed, F. Yoshii, K. Makuuchi, “Radiation vulcanization of acrylonitrile–butadiene rubber with polyfunctional monomers” *React. Funct. Polym.* **2002**, *53*, 173–181.

[3] G. Konstantopoulos, S. Soulis, D. Dragatogiannis, C. Charitidis, “Introduction of a Methodology to Enhance the Stabilization Process of PAN Fibers by Modeling and Advanced Characterization” *Materials* **2020**, *13*, 2749.

[4] Y. Liu, X. Huang, J. Liu, J. Liang, X. Wang, “Structure and tensile properties of carbon fibers based on electron-beam irradiated polyacrylonitrile fibers” *J. Mater. Sci.* **2020**, *55*, 4962–4969.

[5] T. M. Arantes, K. V. Leão, M. I. B. Tavares, A. G. Ferreira, E. Longo, E. R. Camargo, “NMR study of styrene-butadiene rubber (SBR) and TiO2 nanocomposites” *Polym. Test.* **2009**, *28*, 490–494.

[6] K. Masaki, S. Ohkawara, T. Hirano, M. Seno, T. Sato, “Devulcanization of nitrile butadiene rubber in nitrobenzene” *J. Appl. Polym. Sci.* **2004**, *91*, 3342–3353.

[7], “ACS PMI Life Cycle Assessment Tool,” can be found under https://acsgcipr.org/tools/pmi-life-cycle-assessment/(accessed 13 February 2026), **2026**.

[8] H. B. Rose, B. Kosjek, B. M. Armstrong, S. A. Robaire, “Green and sustainable metrics: Charting the course for green-by-design small molecule API synthesis” *Current Research in Green and Sustainable Chemistry* **2022**, *5*, 100324.

[9] C. Kanjilal, B. C. Mitra, S. R. Palit, “Reduction of nitrile end groups in styrene polymers and their subsequent determination by dye partition technique” *Die Makromolekulare Chemie* **1977**, *178*, 1707–1710.

[10] A. E. Finholt, A. C. Bond, H. I. Schlesinger, “Lithium Aluminum Hydride, Aluminum Hydride and Lithium Gallium Hydride, and Some of their Applications in Organic and Inorganic Chemistry ^1^” *J. Am. Chem. Soc.* **1947**, *69*, 1199–1203.
